# Structure of the Acidobacteria homodimeric reaction center bound with cytochrome *c*

**DOI:** 10.1038/s41467-022-35460-6

**Published:** 2022-12-14

**Authors:** Shishang Dong, Guoqiang Huang, Changhui Wang, Jiajia Wang, Sen-Fang Sui, Xiaochun Qin

**Affiliations:** 1 School of Biological Science and Technology, University of Jinan, Jinan, China; 2 State Key Laboratory of Membrane Biology, Beijing Advanced Innovation Center for Structural Biology, School of Life Sciences, Tsinghua University, Beijing, China; 3 Department of Biology, Southern University of Science and Technology, Shenzhen, China

**Keywords:** Photosystem I, Cryoelectron microscopy

## Abstract

Photosynthesis converts light energy to chemical energy to fuel life on earth. Light energy is harvested by antenna pigments and transferred to reaction centers (RCs) to drive the electron transfer (ET) reactions. Here, we present cryo-electron microscopy (cryo-EM) structures of two forms of the RC from the microaerophilic *Chloracidobacterium thermophilum* (CabRC): one containing 10 subunits, including two different cytochromes; and the other possessing two additional subunits, PscB and PscZ. The larger form contained 2 Zn-bacteriochlorophylls, 16 bacteriochlorophylls, 10 chlorophylls, 2 lycopenes, 2 hemes, 3 Fe_4_S_4_ clusters, 12 lipids, 2 Ca^2+^ ions and 6 water molecules, revealing a type I RC with an ET chain involving two hemes and a hybrid antenna containing bacteriochlorophylls and chlorophylls. Our results provide a structural basis for understanding the excitation energy and ET within the CabRC and offer evolutionary insights into the origin and adaptation of photosynthetic RCs.

## Abstract (web summary)

During photosynthesis, light energy is harvested by an antenna systems and delivered to the reaction centers (RCs) for charge separation and electron transfer (ET). The authors report cryo-EM structures of two forms of RC from the microaerophilic *Chloracidobacterium thermophilum* (CabRC), providing a structural basis for ET within the CabRC.

## Introduction

In photosynthesis, light energy is harvested by antenna systems and delivered to reaction centers (RCs) for subsequent charge separation and electron transfer (ET) processes. RCs are classified as type I or type II, depending on whether their terminal electron acceptors are in the form of iron–sulfur clusters or quinones^1,2^. Among the seven existing phyla of photosynthetic bacteria^3,4^, all except the Cyanobacteria use a single RC to drive ET and are characterized by anoxygenic photosynthesis. Among these anoxygenic photosynthetic bacteria, green sulfur bacteria such as *Chlorobium tepidum* (*C. tepidum*, phylum Chlorobi)^5^, heliobacteria such as *Heliomicrobium modesticaldum* (*H. modesticaldum*, phylum Firmicutes)^6,7^, some Chloroflexi such as *Candidatus* Chlorohelix allophototropha (phylum Chloroflexi)^8^ and Acidobacteria such as *Chloracidobacterium thermophilum* (*C*. *thermophilum*, phylum Acidobacteria)^9^ have homodimeric type I RCs. The common ancestor of all RCs, including the heterodimeric photosystem I (PSI)^10,11^ and PSII^12^ found in oxygenic photosynthesis, is thought to have been homodimeric^13–15^. Therefore, it is important to resolve the structures of extant homodimeric RCs to understand the evolution of photosynthesis. The structures of RCs from *H. modesticaldum* (HbRC) and *C. tepidum* (GsbRC) were recently reported^5,6^, contributing considerably to the understanding of the common features of type I RCs, as well as the divergence from the common ancestor. However, the structure of the RC from Acidobacteria is not well understood, and the mechanisms of transfer and conversion of light energy are far from clear.

*C. thermophilum* is a microaerophilic chlorophototroph in the phylum Acidobacteria^16,17^ that is of particular interest because of its unusual mix of chlorophylls *a* (Chls *a*), bacteriochlorophylls *a* (BChls *a*) and Zn-BChls *a*′ (an epimer of BChl *a* that exhibits reversed stereochemistry at the 13^2^ carbon of the tetrapyrrole ring, with Zn^2+^ instead of Mg^2+^ as the central metal)^9,17^. Here, we solve the structures of the RC from *C*. *thermophilum* (CabRC) by single-particle cryo-electron microscopy (cryo-EM) at resolutions of 2.61 Å for a larger form (termed CabRC_L_) and 2.22 Å for a smaller form (termed CabRC_S_). In both structures, two distinctive *c*-type cytochromes (Cyts *c*) are tightly bound, making the anticipated type I RC structures with cytochromes bound be solved. Our research reveals a unique type I RC organization and its cofactor arrangements, providing insight into the evolution of photosystems.

## Results and discussion

### Composition and overall structure

We isolated the CabRC complex from *C*. *thermophilum* cells grown under microoxic conditions by sucrose density gradient centrifugation (Supplementary Fig. 1a) and purified them by size-exclusion chromatography, as described in the “Methods”. We identified the major polypeptides by sodium dodecyl sulfate–polyacrylamide gel electrophoresis (SDS‒PAGE) and mass spectrometry (Supplementary Fig. 1b, c).The absorption spectrum obtained at room temperature showed two peaks in the Q_Y_ band region, a main peak at 812 nm with a shoulder at 825 nm and a secondary peak at 673 nm (Supplementary Fig. 1d), consistent with previous reports^9,17^. The pigment composition is shown in Supplementary Fig. 1e–j. We confirmed the presence of three kinds of (B)Chls *a* (BChl *a*, Zn-BChls *a*′ and Chl *a*) and lycopene by high‑performance liquid chromatography (HPLC), which was consistent with previous reports^9,17,18^.

**Fig. 1:**
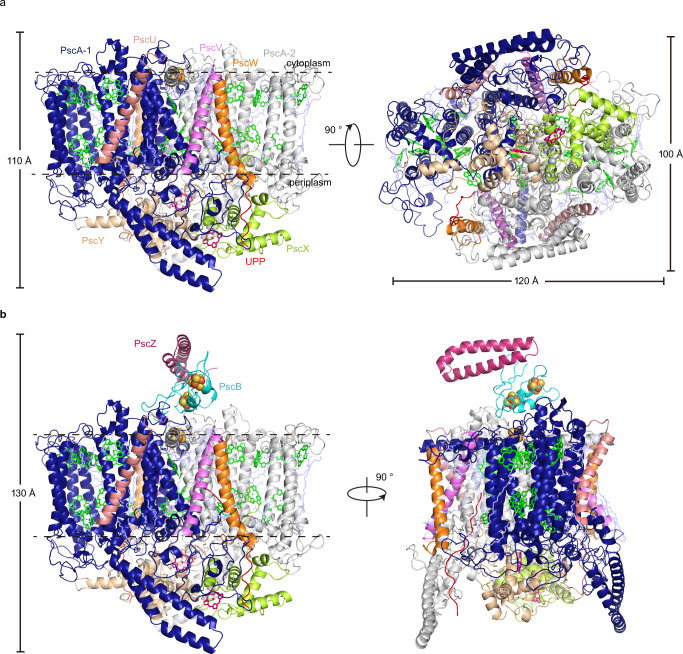
Two structural forms of the CabRC. **a** Structure of the CabRC_S_ complex viewed along the membrane plane from the front (left) and the periplasmic side (right). **b** The structure of the CabRC_L_ complex viewed along the membrane plane from the front (left) and side (right). All subunits are shown as cartoon models of different colors. Carotenoids, lipids and unknown molecules are shown as line models and colored light blue. Only the main tetrapyrrole rings are shown for (B)Chls, and are colored green. Only the main tetrapyrrole rings are shown for heme groups, and are colored hot pink. Fe_4_S_4_ clusters are shown as yellow and oraspheres.

As the molecular weights of CabRC_S_ and CabRC_L_ are too close to allow their biochemical separation, the final purified samples comprised both forms. Additionly, the ratio of CabRC_S_ to CabRC_L_ in different batches differed even following the same purification method. To estimate the approximate ratio of CabRC_S_ to CabRC_L_, we collected about 100 cryo-EM micrographs for each sample batch and judged the ratio from two-dimensional (2D) averages. Based on the primary evaluation, we collected 6626 cyro-EM micrographs from one sample with a high proportion of CabRC_S_ and 1970 cyro-EM micrographs from another sample with a high proportion of CabRC_L_ and used them to determine the structures of CabRC_S_ and CabRC_L_, respectively (Fig. 1, Supplementary Figs. 1–3, and Supplementary Table 1). The CabRC_S_ form (2.22 Å resolution), with dimensions of ~110 Å × 120 Å × 100 Å, contained ten subunits. The largest transmembrane subunit, PscA, formed a homodimer (termed the CabRC core), and three newly identified single-transmembrane subunits (PscU, PscV, and PscW, named in alphabetical order) and an undefined polypeptide (abbreviated as UPP) were located symmetrically on either side of the PscA homodimer, i.e., the front (in view) and back sides in Fig. 1a (left). The UPP spanned the membrane but did not form a helix, which was modeled by polyalanine due to the limited resolution of this polypeptide (Supplementary Fig. 4). Two different Cyts *c* formed a heterodimer, and both were attached to the periplasmic side of the CabRC core, functioning as electron donors (Fig. 1a). We identified them as Cabther_A2183 and Cabther_A2184^19^, which we termed PscX and PscY, respectively, in consideration of the correspondence of their binding sites to that of PscC in the GsbRC despite the lack of substantial sequence similarity with it. We assigned the two monomers within the PscA homodimer as PscA-1 and PscA-2 according to their interactions with the transmembrane parts of PscX and PscY, respectively, to clearly describe the interactions between the CabRC core and the two Cyts *c* (Fig. 1a). The CabRC_L_ form (2.61 Å resolution) possessed two additional extrinsic subunits on the cytoplasmic side, PscB (an electron acceptor protein containing two Fe_4_S_4_ clusters corresponding to PscB in the GsbRC) and PscZ (a newly identified subunit whose binding position is analogous to that of PscD in the GsbRC), forming a larger structure with dimensions of ~130 Å × 120 Å × 100 Å (Fig. 1b and Supplementary Fig. 4). Except for the extra PscB and PscZ, CabRC_L_ was almost the same as CabRC_S._ The sequences of all the subunits are listed in Supplementary Table 2. As CabRC_L_ binds electron donor and acceptor proteins, we describe the characteristics of CabRC based primarily on CabRC_L_. CabRC_L_ contained numerous cofactors, including 16 BChls *a*, 2 Zn-BChls *a*′, 10 Chls *a*, 2 lycopenes, 12 lipids [2 phosphatidyl-ethanolamines (PE), 6 phosphatidyl-N-methylethanolamines (PME) and 4 diacylglycerylhydroxymethyl-N,N,N-trimethylalanines (DGTA)], 3 Fe_4_S_4_ clusters (F_X_, F_A_, and F_B_), 2 heme groups (Heme1 and Heme2), 2 Ca^2+^ ions, 6 water molecules and 32 unknown molecules, in addition to the 12 protein subunits and two UPPs.

Each PscA contained 11 transmembrane helices (TMHs), with a relatively large extramembrane domain on the periplasmic side and a smaller domain on the cytoplasmic side (Supplementary Fig. 5a). As for most other RCs, the TMH region was divided into an N-terminal antenna domain (TMHs 1–6) and a C-terminal ET domain (TMHs 7–11) (Supplementary Fig. 5a). Although structural alignment showed high conservation of the TMH regions among different RCs, a unique region of ~205 amino acids (N440–H644) in the CabRC was inserted between TMH 7 and TMH 8, leading to a wing-like structure protruding into the periplasmic space (Supplementary Fig. 5b–g). The existence of two copies each of PscU, PscV and PscW in the CabRC results in a larger number of subunits surrounding the core than in GsbRC and HbRC, but fewer than in PSI or PSII. To compare the locations of the PscU, PscV and PscW peripheral subunits in the CabRC with subunits surrounding the core in other RCs, we defined four regions in the CabRC. Region I (or IV) was located in the peripheral region of helix 7 from PscA1 (or PscA2) and helixes 1, 2, 10, and 11 from PscA2 (or PscA1), and region II (or III) was located in the peripheral region of helixes 4, 5, 6, 7 and 8 from PscA1 (or PscA2) (viewed from the cytoplasmic side). Thus, PscW and PscV subunits were located in regions I and IV, and PscU subunits were located in regions II and III (Fig. 1a and Supplementary Fig. 6a), which was more similar to the distribution of the low-molecular-weight proteins in PSII^20^ (Supplementary Fig. 6e) than to the distribution of surrounding subunits in the HbRC^6^, GsbRC^21,22^ and PSI^10^ (Supplementary Fig. 6b–d). This different pattern would be an important point about divergence between the CabRC and other type I RCs (especially GsbRC) during their evolution from a common ancestor. For the CabRC, these peripheral subunits increased its size and were just as likely to form an interface between the RC and other proteins in the membrane. PscZ, consisting of 60 amino acids (M1–L60) and forming a two-helix structure, resided at the cytoplasmic side and connected to PscB through hydrophobic interactions, hydrogen bonds, and salt bridges (Fig. 1b and Supplementary Fig. 7d, e). The location of PscZ in the CabRC corresponded to PscD in the GsbRC or PsaD in PSI, even though there were no similarities in primary sequences and spatial structures between them (Supplementary Fig. 7a–c); therefore, PscZ may stabilize the electron acceptors in the CabRC in a manner similar to PscD in GsbRC or PsaD in PSI^5,10,11^.

### ET chain is extended by two heme groups

**Fig. 2:**
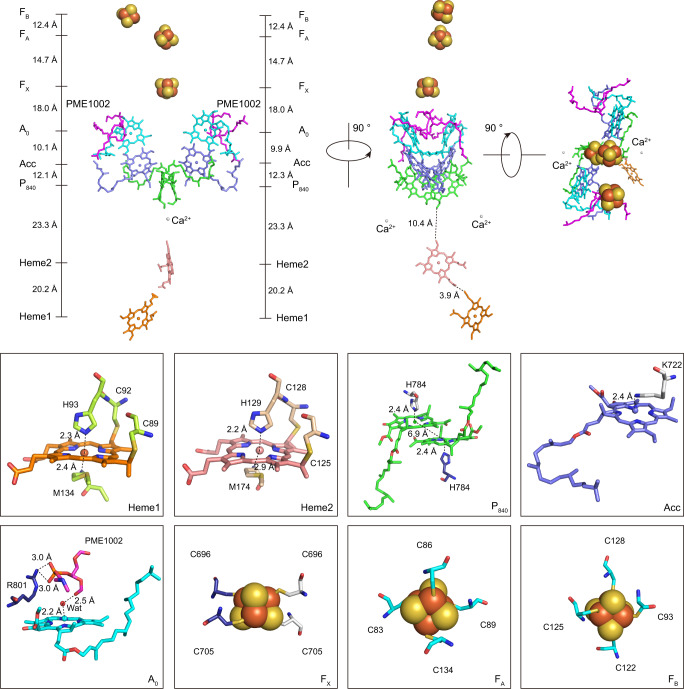
Cofactor arrangement of the ETC in the CabRC viewed along the membrane plane and from the periplasmic side. The center-to-center distances between the cofactors (black dotted lines) are given in Å. The boxed areas show the coordinating environments for all cofactors in the ETC. The cofactors Heme1, Heme2, P_840_, Acc, A_0_, and Fe_4_S_4_ (F_X_, F_A_, and F_B_) are colored orange, salmon red, green, light blue, cyan, and orange/yellow, respectively. The backbone carbon atoms of PME1002 are colored magenta. Calcium ions (Ca^2+^) and water molecules (Wat) are shown as spheres and colored gray and red, respectively. For clarity, the tail of PME1002 is omitted in the boxed areas.

Our structure showed a long-distance ET chain (ETC) containing two heme groups serving as secondary electron donors, one special pair of Zn-BChls *a*′ serving as the primary electron donor (P_840_), two Chls *a* serving as the accessory (B)Chl *a* (Acc), and a series of electron acceptors: i.e., another two Chls *a* serving as the primary acceptor (A_0_) and three Fe_4_S_4_ clusters (F_X_, F_A_, and F_B_) (Fig. 2).

We observed five distinctive features in the ETC. First, based on spectroscopic studies^23,24^, P_840_ was assigned as a pair of Zn-BChls *a*′ separated by an interplane distance of 6.9 Å, wherein Zn^2+^ was coordinated by H784/PscA at a distance of 2.4 Å, which is shorter than the distance observed in the GsbRC (2.9 Å)^5^. Among the known RCs, the CabRC was the only one in which the metal ions are differed between the cofactors in the special pair and antenna (B)Chls. Second, in contrast to the suggestion that each Acc is a BChl *a* (or Zn-BChl *a*′) in the CabRC^17^, the cryo-EM map unambiguously identified Acc and A_0_ as Chls *a* (Supplementary Fig. 8). This represents a difference from the HbRC, in which each Acc is a BChl *g* molecule^5^, and from the GsbRC, for which there is no definitive conclusion because the local resolution map of the GsbRC cryo-EM structure was too low in resolution to differentiate between BChl *a* and Chl *a*^5^, although its Acc cofactors were assigned as Chls *a* based on previous spectroscopic data^25^. Despite the variety of Acc among these RCs, the A_0_ positions were all invariably occupied by Chl *a* or a Chl *a* devirative. Third, in the CabRC, we found no densities corresponding to the two phylloquinones present in the PSI ETC; however, we observed another two densities near the A_0_ sites. Similar features were also seen in the GsbRC and HbRC structures, and the unmodelled density in the GsbRC structure^5^ was recently assigned as a phosphatidylglycerol^26^, while that in the HbRC was assigned as an undefined molecule with one phosphate headgroup and one single isoprenoid tail^6,26^. In the CabRC, we modeled the analogous density as a PME molecule (PME1002) (Supplementary Fig. 8), a type of lipid that has been reported to be present in *C*. *thermophilum* cells^18^. The phosphate group of PME1002 interacted with R801/PscA via ionic bonds, and its carbonyl group formed a hydrogen bond with a water molecule coordinated to A_0_ (Fig. 2), which is consistent with the interaction involving phosphatidylglycerol in the GsbRC^26^. Most importantly, PME1002 was very close to the position of phylloquinone molecules in PSI, suggesting that it may indirectly affect the ET process via two pairs of interactions (PME1002-R801/PscA and PME1002-water-A_0_). In addition, the edge-to-edge distance between A_0_ and F_X_ (11.5 Å) was very similar to those in the HbRC and GsbRC, and shorter than the distance of ~14.3 Å in the heterodimer PSI structure, which was close enough for forward ET from A_0_ to F_X_. Therefore, the CabRC structure reinforced a view that the homodiemric type I RC ET from A_0_ to F_X_ may not require quinone or menaquinone molecules^6^, which is very different from PSI. Fourth, this is the first time that cytochrome subunits have been resolved in the ETC of a type I RC. The structure pinpointed ET in series from Heme1 to Heme2 and then to P_840_, with edge-to-edge distances of 3.9 Å between Heme1 and Heme2 and 10.4 Å between Heme2 and Zn-BChls *a*′. This arrangement differed from a proposed model for the GsbRC, in which two identical heme groups donate electrons to P_840_ in parallel^27–29^, but was similar to structures found in some purple bacterial RCs, in which *c*-type cytochromes serve as electron donors except that their heme groups are derived from a single peptide^30,31^. Finally, the locations of F_A_ and F_B_ in the CabRC were opposite those in the PSI ETC (see below).

**Fig. 3:**
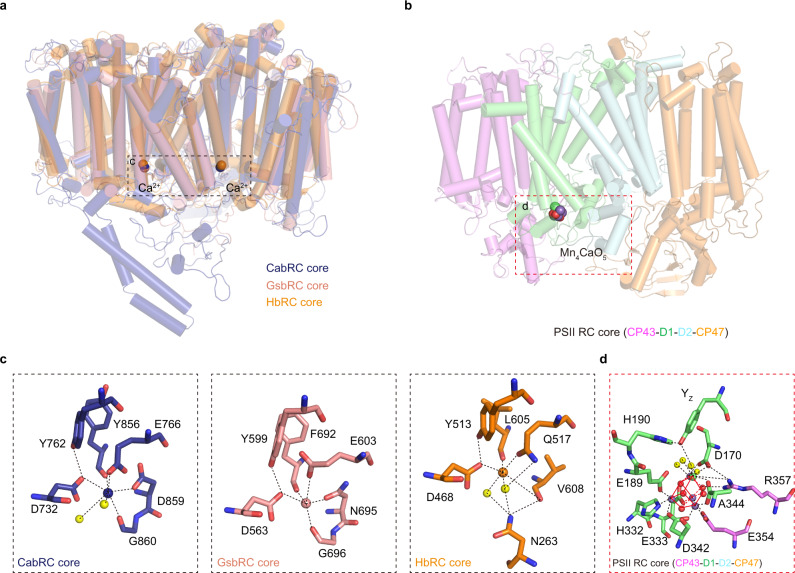
Comparison of the Ca^2+^-binding environments in homodimeric type I RCs and the Mn_4_CaO_5_-binding environment in PSII. **a** Superposition of the CabRC core with the GsbRC core (PscA homodimer; PDB code: 6M32) and the HbRC core (PshA homodimer; PDB code: 5V8 K) to show the positions of their Ca^2+^-binding sites. **b** The position of the Mn_4_CaO_5_ cluster in the PSII RC core (CP43-D1-D2-CP47; PDB code: 3WU2). **c** Magnified views of the Ca^2+^-binding environments in the CabRC core, GsbRC core, and HbRC core. Calcium ions in the CabRC core, GsbRC core, and HbRC core are colored blue, salmon red, and brown, respectively. **d** Magnified views of the Mn_4_CaO_5_-binding environment in PSII. Calcium ions, Mn_4_CaO_5_, and water molecules are shown as spheres, and the Ca^2+^-coordinating residues are shown as sticks; water molecules, Mn ions, and oxygen atoms are colored yellow, purple, and red, respectively; interactions are indicated by black dotted lines.

In addition, two Ca^2+^ ions were located on the electron donor side of the CabRC, each coordinated by five residues (D732, Y762, Y856, D859, and G860 of PscA) and two water molecules. The GsbRC and HbRC contain analogous symmetrical Ca^2+^-binding sites, and in all cases, one of these was in a similar position to the Mn_4_CaO_5_ cluster-binding site in PSII^5,12^ (Figs. 2 and 3). A structural comparison between the Ca^2+^-binding sites in the three homodimeric RCs and the Mn_4_CaO_5_ cluster of PSII revealed both similarities and discrepancies. First, two conserved residues (D732 and Y762 in the CabRC, D563, and Y599 in the GsbRC, and D468 and Y513 in the HbRC) were observed at a position that overlaps with Y_Z_ in PSII. Second, C-terminus residues coordinated to the Ca^2+^ or Mn_4_CaO_5_ cluster: Y856, D859, and G860 in PscA/CabRC; F692, N695, and G696 in PscA/GsbRC; L605 and V608 in PshA/HbRC; and D342 and A344 in D1/PSII. Third, no residue connected the water molecules around the Ca^2+^ in the CabRC, whereas in the HbRC and PSII, the water molecules are linked by their respective residues located in the extrinsic domain between TMH 5 and TMH 6^26^. These structural similarities imply that the local protein environments accommodating the Mn_4_CaO_5_ cluster may already have existed before the divergence of type I and type II RCs. Our structure not only confirms the Ca^2+^-binding sites shared by all three homodimeric type I RCs but also provides insight into the evolution of the Mn_4_CaO_5_ cluster^32,33^. Although further research is needed to determine whether and how Ca^2+^ exerts a unique function in ET, it seemed possible that Ca^2+^ plays a structural role in maintaining ET from heme to the special pair; therefore, we analyzed the two Ca^2+^-binding sites in the subsection of the ETC.

### Electron donor proteins and their interactions with the CabRC core

Due to the diversity of chlorophototrophs, the electron donors vary greatly among different RCs. In oxygenic phototrophs, a water soluble Cyt *c*_6_ or plastocyanin transfers electrons from Cyt *b*_6_*f* to PSI^34^. In some anoxygenic photosynthetic bacteria with type II RCs, a tetraheme Cyt *c* subunit binds to the RCs and mediate between the diffusing electron carrier and the primary electron donor^30,31,35^. For anoxygenic photosynthetic bacteria with a type I RC, although it has been suggested that two identical Cyt *c* subunits containing TMHs, called PscC, function as electron donors in the GsbRC^5,29^, structures of the stable RC complex and the proposed electron donors have not been determined. Thus, visualization of the arrangements of the electron donor proteins is needed.

**Fig. 4:**
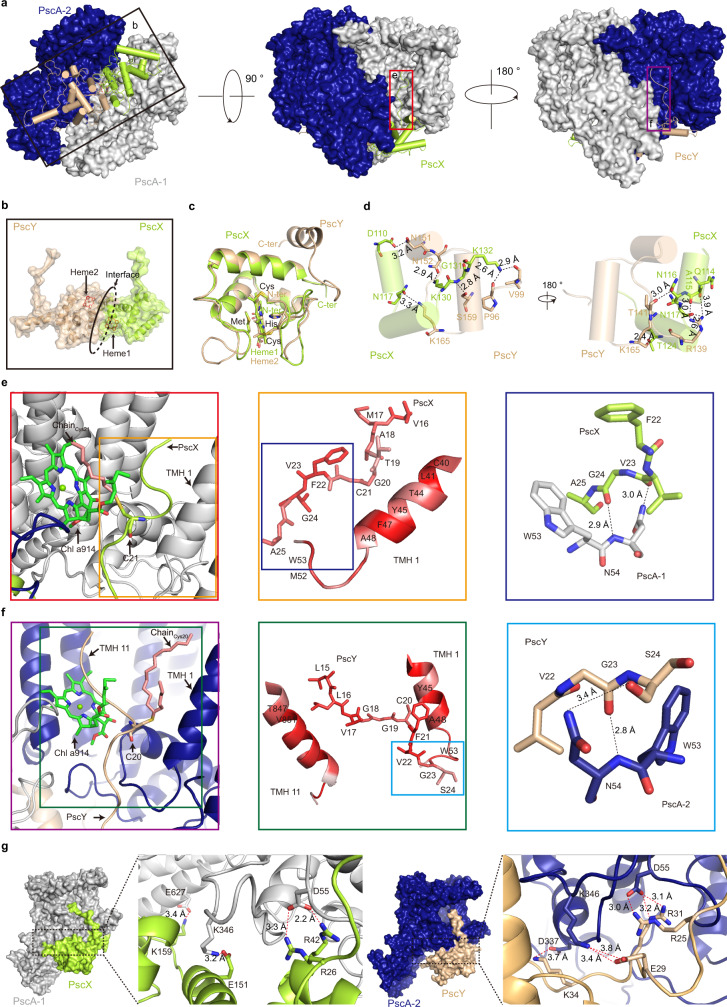
Structures of PscX and PscY and their interactions with the CabRC core. **a** The PscX‒PscY subcomplex interacts with the CabRC core at different sites. Details in the square boxes are presented in (**b**), (**e**), and (**f**). **b** Arrangement of the PscX‒PscY subcomplex in the CabRC. The interface of the two proteins is indicated by a black ellipse. **c** Superposition of the monoheme binding domains of PscX and PscY. **d** Hydrogen bonds (black dotted lines) formed between residues at the interface of PscX and PscY. **e**, **f** Magnified views of the interactions between PscX or PscY and the CabRC core in the transmembrane region. The brown and green boxes (in left panels) are enlarged, and the enlargements (center) show the main hydrophobic interactions between PscX or PscY and the CabRC core; the hydrophobicity is colored red. The dark blue and cyan boxes are further enlarged, and the enlargements (right) show the hydrogen bonds are indicated by black dotted lines. **g** Magnified views of the interactions between PscX or PscY and the CabRC core in the periplasmic region. All salt bridges are indicated by red dotted lines.

In our structures, we modeled two mono-heme electron donor proteins, PscX and PscY, on the periplasmic side of the membrane (Fig. 4a) as a heterodimeric structure (Fig. 4b). The superposition of the two monoheme binding domains had a root mean square deviation of 0.5 Å (Fig. 4c), indicating a high degree of structural similarity despite sharing <25% sequence identity. PscX and PscY formed a large contact surface area (Fig. 4d) through face-to-face interactions, mostly through 11 hydrogen bonds (Fig. 4d and Supplementary Table 3). At this interface, Heme1 and Heme2 were located in the deep grooves created by their respective polypeptides (Fig. 4b, c), which may reduce their exposure to oxygen to prevent oxidation.

PscX and PscY interacted with the CabRC core at two regions. In the transmembrane region, we observed three types of interactions. (1) The N-terminal loop domain (residues 16–25 in PscX and residues 15–24 in PscY) inserted into the membrane mainly through hydrogen bonds and hydrophobic interactions with TMH 1 of PscA-1 (Fig. 4e) and TMHs 1 and 11 of PscA-2 (Fig. 4f). (2) Two cysteine residues, C21/PscX and C20/PscY, may be subject to post-translational modifications that provide a hydrophobic anchor to the RC core. Each cysteine residue was linked to an unknown density, and we modeled the density linked with C21/PscX as a short hydrocarbon chain (termed Chain_Cys21_, Fig. 4e, left) and that linked with C20/PscY as a long hydrocarbon chain (termed Chain_Cys20_, Fig. 4f, left). (3) The tail of one Chl *a*914 embraced Chain_Cys21_, while the tail of the other Chl *a*914 held the N-terminal loop of PscY, revealing a discrepancy in pigment–protein interactions between the two Chl *a*914 molecules, and therefore breaking the symmetry of the pigment microenvironments in the homodimeric RC. In the membrane-extrinsic region, each Cyt *c* associated with PscA-1 and PscA-2 residues in the periplasmic region through numerous hydrogen bonds (Supplementary Table 3) and a few salt bridges (Fig. 4g). We observed these salt bridges at three residues (E267, K346, and D55) of PscA-1 and another three residues (D337, K346, and D55) of PscA-2 with corresponding residues of PscX and PscY, demonstrating that K346 and D55 in either PscA monomer within the homodimeric CabRC play an important role despite the asymmetry of the heterodimeric PscX‒PscY subcomplex. Thus, these close connections maintain the stable binding of the two Cyts *c* and ensure that ET occurs from the heme groups to the photooxidized P_840_.

### Electron acceptor protein and its interactions with the CabRC core

**Fig. 5:**
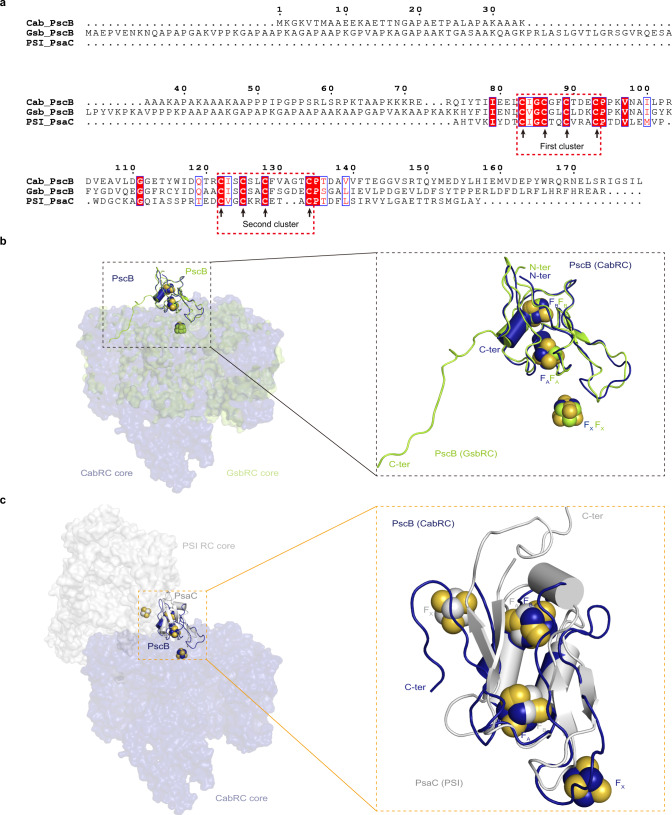
Comparison of the F_A_-F_B_ arrangements in membrane-extrinsic electron acceptors. **a** Comparison of the primary sequences among three membrane-extrinsic electron acceptors. All conserved residues coordinated to the Fe_4_S_4_ cluster are indicated with black arrows. This figure was generated using ESPrint 3^54^. **b** Arrangement of F_X_, F_A_, and F_B_ in the CabRC and GsbRC, based on the superposition of their PscB subunits. **c** Arrangement of F_X_, F_A_, and F_B_ in the CabRC and PSI based on the superposition of PscB/CabRC and PsaC/PSI. PDB codes: GsbRC, 6M32; PSI, 1JB0.

**Fig. 6:**
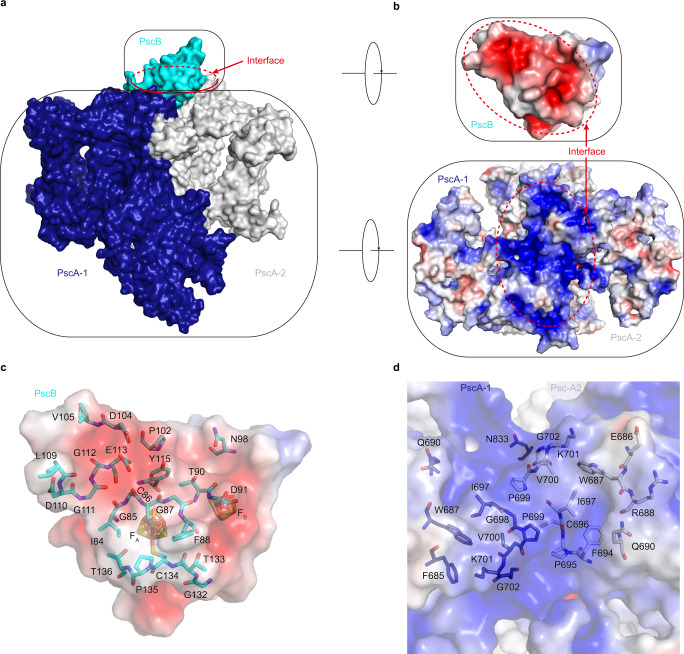
Interactions between PscB and the CabRC core. **a** Arrangement of PscB and the CabRC core. The interface is indicated by red ellipse. **b** Electrostatic potential (calculated with APBS tools^55^) analysis of the binding interface between PscB and the CabRC core. Positive potential is shown in blue (+5 kT/*e*) and negative potential in red (−5 kT/*e*). Close-up views of the surfaces of PscB (**c**) and the CabRC core (**d**). All residues on the interfaces at a distance of 5 Å or less are shown with sticks.

PscB of the CabRC is an extrinsic electron acceptor protein with a function similar to those of PscB of the GsbRC and PsaC of PSI^5,10,36^. Sequence alignment between PscB/CabRC, PscB/GsbRC and PsaC/PSI showed low sequence similarity, reflecting billions of years of evolutionary divergence; however, all of them share a CXXCXXCXXXC cluster (the first cluster) and a CXXCXXC(XX)XXXC cluster (the second cluster) used for binding two Fe_4_S_4_ clusters (Fig. 5a). We identified the positions of F_A_ and F_B_ in PscB/CabRC based on sequence alignment with PscB/GsbRC, in which F_A_ was the proximal cluster (a Fe_4_S_4_ cluster, coordinating with the first three cysteines of the first cluster and the fourth cysteine of the second cluster), and F_B_ was the distal cluster (a Fe_4_S_4_ cluster, coordinating with the first three cysteines of the second cluster and the fourth cysteine of the first cluster) (Figs. 2, 5a). Based on the structural superposition with PscB/GsbRC and PsaC/PSI, the F_A_–F_B_ arrangement in PscB of the CabRC was very similar to that in the GsbRC; unexpectedly, however, it appeared to be switched relative to that in PSI (Fig. 5b, c). This means that in PsaC of PSI, F_A_ is coordinated by the first three cysteines of the second cluster and the fourth cysteine of the first cluster, and that F_B_ is coordinated by the first three cysteines of the first cluster and the fourth cysteine of the second cluster, which is reversed relative to the arrangement in PscB of the CabRC and GsbRC. These characteristics suggest that, compared with the PsaC subunit in PSI, the acceptor proteins binding F_A_–F_B_ in the CabRC and GsbRC are nearly upside down when binding with their respective RC cores. Further analysis revealed that the PscB/CabRC binds to the surface of its RC core mainly through the weak electrostatic interactions between the negatively charged patch on the PscB side and the positively charged patch on the CabRC core side (Fig. 6), as well as one weak hydrogen bond between D91/PscB and K701/PscA-2 (Supplementary Table 4), which is consistent with the fact that the PscB and PscZ subunits in the CabRC are easily removed during purification^9^. We observed similar electrostatic and hydrogen-bonding interactions between PscB/GsbRC and its RC core, but in this case, the electrostatic interactions were weaker and the hydrogen-bonding interactions stronger (Supplementary Table 4). In PSI, in addition to fixation by PsaD and PsaE, PsaC binds to the PsaA–PsaB subcomplex mainly due to the additional hydrogen bonds and salt bridges, rather than the weak electrostatic interactions between PsaC and PsaA–PsaB subcomplex^10^ (Supplementary Table 4). Thus, the mode and strength of binding of PscB to the CabRC core are differed from those of PscB in the GsbRC or PsaC in PsaA–PsaB subcomplex, reflecting the evolutionary changes in the interactions between electron acceptors and their RCs among various type I photosystems.

Another common feature between the CabRC and the GsbRC is that both link Fenna–Matthews–Olson proteins (FMOs)-chlorosomes serving as light-harvesting antennas on the cytoplasmic side, which may influence PscB and ferredoxin binding^27^. In contrast, PSI from cyanobacteria binds membrane-extrinsic soluble phycobilisomes as antennas, and PSI from algae and land plants does not bind any membrane-extrinsic antennas. However, whether the reversed binding of the acceptor proteins and the resulting F_A_–F_B_ arrangement is related to the divergence of light-harvesting antennas deserves further investigation.

### Hybrid light-harvesting system

**Fig. 7:**
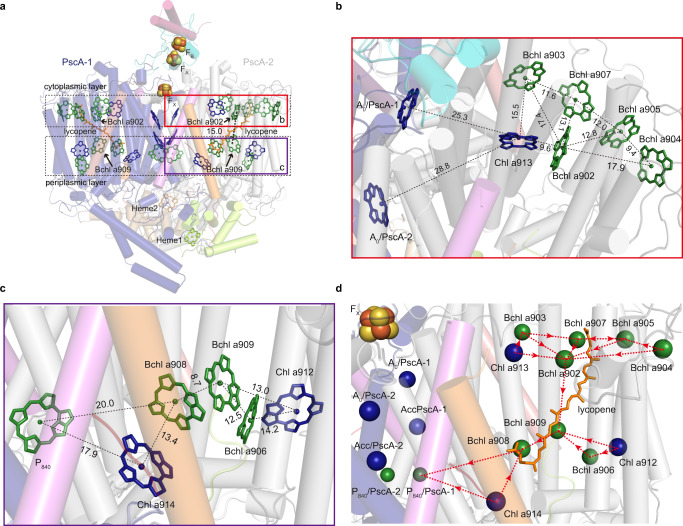
Distribution of (B)Chls *a* and lycopenes and possible ET pathways in the CabRC. **a** Pigments in the CabRC are arranged into the cytoplasmic layer (closer to the cytoplasmic side) and periplasmic layer (closer to the periplasmic side). Chls *a* are colored dark blue, Zn-BChls *a*′ and BChls *a* are colored green, and lycopenes are colored brown. Arrangement of pigments at the cytoplasmic layer (**b**) and periplasmic layer (**c**), with Mg^2+^-to-Mg^2+^ distances given in Å. **d** Possible ET pathways in the CabRC. The directions of ET are indicated with red dotted arrows. Chls *a* and BChls *a* are represented by dark blue and green spheres, respectively.

The most striking feature of the antenna pigments in the CabRC was the coexistence of BChls and Chls, making it possible to harvest both visible and far-red light. In contrast, other RCs can use only BChls or Chls; therefore, we refer to the light-harvesting system of the CabRC as a hybrid. The resolution of our structure was sufficiently high to distinguish between BChls *a* and Chls *a* (Supplementary Fig. 8), providing a rare opportunity to investigate how energy is transferred in a hybrid pigment network. Our CabRC structure unequivocally showed that 28 (B)Chls *a* (16 BChls *a*, 10 Chls *a* and two Zn-BChls *a*′) are distributed within cytoplasmic and periplasmic layers, which differs somewhat from the ratio of BChls *a*:Chls *a*:Zn-BChls *a*′ (12.8:8.0:2.0 or 32:24:4)^9,17^. In addition to the ETC cofactors, each PscA contained one Chl *a*913 and five BChls *a* (902–905 and 907) in the cytoplasmic layer, and two Chls *a* (912 and 914) and three BChls *a* (906, 908 and 909) in the periplasmic layer, and the closest Mg^2+^-to-Mg^2+^ distance between the two layers was 15.0 Å between BChl *a*902 and BChl *a*909 (Fig. 7), which is considered the main excitation energy transfer pathway between the two layers.

As BChl *a* has a much lower energy level than Chl *a*, energy transfer from BChl *a* to Chl *a* is thermodynamically a steep uphill process^37^. However, the excitation energy can be transferred from Chl *a* to BChl *a* within a relatively close distance^38^. In the cytoplasmic layer, Chl *a*913 was the closest (B)Chl *a* to ET chain A_0_ (Chl *a*911), with a Mg^2+^-to-Mg^2+^ distance of 25.3 Å, Half of BChl *a*902 was surrounded by four BChls *a* (903, 907, 905, and 904) and Chl *a*913, and the Mg^2+^-to-Mg^2+^ distance from BChl *a*902 was 17.4 Å to BChl *a*903, 13.1 Å to BChl *a*907, 12.8 Å to BChl *a*905, 17.9 Å to BChl *a*904, and only 9.6 Å to Chl *a*913. The Mg^2+^-to-Mg^2+^ distances between other adjacent (B)Chls *a* were also short (Fig. 7b), with an average of only 12.1 Å. Therefore, based on the distances, energy captured by cytoplasmic layer (B)Chls *a* can be transferred to BChl *a*902 and then delivered to BChl *a*909 in the periplasmic layer (Fig. 7d). In the periplasmic layer, Chl *a*914 was the closest (B)Chl *a* to P_840_ (901), with a Mg^2+^-to-Mg^2+^ distance of 17.9 Å, which was 2.1 Å shorter than the distance between P_840_ (901) and BChl *a*908. BChl *a*909 was close to the proximal BChl *a*908 and the distal (B)Chls *a* (912 and 906), with Mg^2+^-to-Mg^2+^ distances of 8.7, 13.0, and 12.5 Å, respectively (Fig. 7c). Thus, BChl *a*908 likely obtain energy from BChl *a*909 and Chl *a*914 and transfer it to P_840_. It should be noted that direct energy transfer from Chl *a*914 to P_840_ may occur simultaneously because of their close Mg^2+^-to-Mg^2+^ proximity (17.9 Å) (Fig. 7c, d). Overall, the pigment distribution of the CabRC facilitates efficient excitation energy transfer from the antenna pigments to P_840_.

We identified a pair of conserved antenna (B)Chls *a* in the GsbRC (Bchls *a*814), the HbRC (BChl *g*1024), PSI (Chl *a*1136/PsaA‒Chl *a*1235/PsaB) and PSII (Chl_Z_‒Chl_D_) structures, but not in the CabRC structure (Supplementary Fig. 9a). This result might be explained by a substitution of the histidine in the GsbRC, HbRC, PSI and PSII with tryptophan in the CabRC, obliterating the site for coordinating Mg^2+^ in the CabRC, which is consistent with a previous observation that histidine is not always conserved among different RC core subunits^26^. We speculated that the loss of the two peripheral (B)Chls *a* in the CabRC allows the recruitment of proteins to create a larger antenna size for light harvesting. As a microaerophilic chlorophototroph, *C*. *thermophilum* express a unique carotenoid-binding protein, which may act as an intrinsic, peripheral antenna for light harvesting, oxygen tolerance, and photoprotection^9^.

Furthermore, we identified one transmembrane lycopene molecule in each PscA. Lycopene is a noncyclic carotenoid, and one of its termini inserted into the space enclosed by BChl *a*902, BChl *a*905 and BChl *a*907 in the cytoplasmic layer, with the other terminus extended to the gap between BChl *a*908 and Chl *a*914 in the periplasmic layer (Fig. 7a). This transmembrane lycopene can absorb and transfer light energy to its adjacent (B)Chls *a*, but it does not transfer energy between the two layers as this would require a steep uphill step. In contrast, at the corresponding site, the GsbRC binds a half-transmembrane chlorobactene (F26)^5^, and the HbRC binds a short-chain carotenoid (4,4′-diaponeurosporene) nearly parallel to the membrane^6^. In addition, lycopene is an effective singlet oxygen scavenger^39^, and our results suggested that the transmembrane lycopene in the CabRC is the most powerful among the three RCs to prevent potentially harmful singlet oxygen formation, consistent with its microaerophilic environment. However, the GsbRC binds one additional carotenoid, a glycosylated carotenoid (F39), than the CabRC within the peripheral region of the RC, and similarly, a *β*-carotene molecule is present in an analogous location in PSII^26^. As this region was named the “L” region to indicate that it is the lipid- and carotenoid-containing region conserved in the GsbRC and PSII but not in the HbRC or PSI^26^, we checked the distribution of CabRC lipids (Supplementary Fig. 9b). The CabRC also contained two lipid molecules (PE1004 and PME1001), indicating a partially conserved feature among the CabRC, GsbRC, and PSII. The diversity of the structure and location of carotenoids and lipids in these homodimeric RCs reflects a specific adaptation to different environments.

In conclusion, we solved the high-resolution structures of the CabRC, which is the third homodimeric type I RC structure reported, following the HbRC and GsbRC. The CabRC structure itself and some comparisons among them revealed a number of unique features of the CabRC that not only are important for understanding light energy and electron transfer reactions but also provide insight into the evolutionary processes of extant photosystems.

## Methods

### Purification of the CabRC complex

*Chloracidobacterium thermophilum*^16^ cells (ATCC BAA-2647) were grown in 1 L of CTM medium for 2 weeks under 40 μmol photons m^−2^ s^−1^ provided by a tungsten lamp at 52 °C^40^. In the following procedures, ascorbic acid was added to all buffers to avoid oxidation of the CabRC, and all steps were performed under green light. The cells were harvested by centrifugation for 15 min at 8000 × *g*, and the pellet (6 g, wet weight) was resuspended in 15 mL of lysis buffer (20 mM Tris-HCl, pH 7.6) supplemented with 0.1 mg/mL DNase I and 10 mM ascorbic acid^5^. The cells were disrupted by sonication for 15 min in an ice bath and then homogenized five times at 2000 bar with a low-temperature ultra-high-pressure cell disrupter (JNBIO). The lysate was centrifuged for 10 min at 8000 × *g* to remove cell debris and unbroken cells. The supernatant was then centrifuged for 90 min at 220,000 × *g*. The resulting pellet containing membranes was collected and solubilized by 3% (w/v) Triton X-100 in an ice bath for 70 min. Unsolubilized materials were removed by centrifugation for 60 min at 57,750 × *g*, and the supernatant containing the CabRC was collected and further purified by a linear gradient of sucrose from 300 to 900 mM at 243,500 × *g*. After ultracentrifugation for 16 h at 4 °C, three bands were separated, among which B1 contained free pigments, B2 mainly contained crude CabRC samples, and B3 contained the other components including chlorosomes (Supplementary Fig. 1a, b). Sample B2 was concentrated and purified with a Superose 6 Increase 10/300 GL column (GE) equilibrated with storage buffer (20 mM Tris-HCl, pH 7.6, 10 mM ascorbic acid, and 0.03% Triton X-100). The purified CabRC samples were pooled and concentrated to 60.0 mg/mL for use in the subsequent experiments. All main subunits were identified by mass spectrometry (Supplementary Fig. 1c). Source data are provided in the Source Data file.

### HPLC analyses

All three kinds of pigments (BChl *a*, Zn-BChl *a*′ and Chl *a*) and lycopene were extracted from the purified CabRC samples in acetone:methanol (7:2, by volume) and analyzed by HPLC^18^. The extracted samples were analyzed on an analytical C18 column (3.0 mm by 25 cm) (Thermo Scientific). Solvent A (methanol:acetonitrile: water, 42:33:25 by volume) and solvent B (methanol:acetonitrile:ethyl acetate, 50:20:30 by volume) were used for elution. The program was performed as follows: at the time of injection, 30% B; a linear increase from 30% B to 100% B in 52 min; constant 100% B for 6 min; and a return to 30% B in 2 min. The column temperature was 35 °C, and the flow rate was 1.0 ml/min^5^. BChl *a* and Zn-BChl *a*′ were monitored at 770 nm, Chl *a* was monitored at 665 nm, and lycopene was monitored at 491 nm. All elution profiles and absorption spectra features are shown in Supplementary Fig. 1e–j.

### Cryo-EM sample preparation and data collection

A 4 μL aliquot of the CabRC sample (6.0 mg/mL) was applied to a holey carbon grid (Quantifoil Au grid, R1.2/1.3, 400 mesh) and plunged into liquid ethane cooled by liquid nitrogen using an FEI Vitrobot Mark IV. The parameters used for plunge freezing were as follows: blotting time of 4 s, blotting force level of 0, waiting time of 15 s, chamber humidity of 100% and temperature of 8 °C. Cryo-EM sample screening was performed using a Tecnai Arctica or Talos Arctica operated at 200 kV and equipped with an FEI K2 camera. Cryo-EM micrographs used for high-resolution structure determination were collected with a Titan Krios Microscope (Thermo Fisher Scientific) operated at 300 keV and equipped with a K3 Summit direct electron detector (Gatan) and a GIF Quantum energy filter (Gatan). The cryo-EM images were automatically collected using AutoEMation^41^ with a slit width of 20 eV on the energy filter and a preset defocus range of −1.5 to −1.0 μm in super-resolution mode at a nominal magnification of 81,000×, corresponding to a calibrated pixel size of 0.541 Å. Finally, 6626 micrographs for the CabRC_S_ complex and 1970 micrographs for the CabRC_L_ complex were recorded. Each stack of 32 frames was exposed for 8 s, and the total dose was approximately 50 e^–^/Å^2^.

### Cryo-EM image processing

The movie stacks were motion-corrected with MotionCor2^42^, binned to a pixel size of 1.0825 Å, and then imported into cryoSPARC^43^. Contrast transfer function (CTF) parameters were determined by patch CTF estimation in cryoSPARC. For the CabRC_S_ complex, 43,854 particles were auto-picked from 100 micrographs using a blob picker and subjected to 2D classification. Then, 7272 particles were selected as the training dataset for Topaz^44^. Finally, 1,523,482 particles were auto-picked from 6626 micrographs by Topaz and extracted from non-dose-weighted micrographs with a box size of 56 pixels (binned 4×, 4.33 Å/pixel). After 2D classification, 1,298,021 particles were selected for ab initio reconstruction to generate the initial model, and only one class with 647,088 particles was selected for non-uniform refinement^45^, which yielded a 9.39 Å resolution map. The particles were re-extracted with a pixel size of 2.165 Å (binned 2×, box size of 128 pixels) and then 1.0825 Å (binned 1×, box size of 220 pixels). A round of heterogeneous refinement with three references was performed to screen bad particles, and 490,075 particles were kept for further processing. After CTF refinement and non-uniform refinement, particles were re-extracted from dose-weighted micrographs. Finally, a 2.22 Å resolution map was obtained after non-uniform refinement. These particles were then imported in RELION3.1^46,47^ by using pyem package (10.5281/zenodo.3576630), which also yielded a 2.22 Å resolution map after 3D auto-refinement and post-processing.

For the CabRC_L_ complex, 53,383 particles were auto-picked from 100 micrographs by a blob picker, and subjected to 2D classification. Then, eight 2D-averaged classes with 8520 particles were selected as templates for template-based auto-picking. In total, 905,788 particles were auto-picked from 1970 micrographs and extracted from non-dose-weighted micrographs with a box size of 56 pixels (binned 4×, 4.33 Å/pixel). After 2D classification, 767,477 particles were selected and subjected to ab initio reconstruction to generate the initial models. Subsequently, one class with 374,584 particles was selected for non-uniform refinement, which yielded a 9.09 Å resolution map. Then, particles were re-extracted at a pixel size of 1.0825 Å with a box size of 220 pixels. After performing particle re-extraction from dose-weighted micrographs, CTF refinement and non-uniform refinement, a 2.36 Å resolution map was obtained. However, the density of the cytoplasmic region is much worse than that of the core region, and many strategies were used to separate particles with clear density from other particles in the cytoplasmic region. First, 374,584 particles were imported into RELION3.1, and two rounds of masked 3D classification were performed with a local mask covering the cytoplasmic region and a part of the core region to help alignment. After that, 146,118 particles with clear cytoplasmic region density were selected for 3D auto-refinement. Second, particle subtraction was performed with a mask including the region outside the cytoplasmic region to be subtracted, and the subtracted particles were re-centered and subjected to 2D classification. Classes with clear density of the cytoplasmic region could be distinguished from 2D averages, and 52,612 particles were selected. The remaining particle coordinates were imported into cryoSPARC, and whole particles, including the core region and the cytoplasmic region, were re-extracted with a box size of 220 pixels. After non-uniform refinement, a 2.61 Å global resolution map was obtained with local resolutions of the cytoplasmic region ranging from ~3.5 to ~5.0 Å.

All reported resolutions were estimated based on the gold-standard FSC 0.143 criterion. Local resolution maps were calculated by RELION 3.1/3.0. Flowcharts of cryo-EM data processing are shown in Supplementary Figs. 2 and 3.

### Modeling and refinement

An atomic model of the CabRC_S_ complex was de novo built using an improved version of the A^2^-Net deep neural network method^48^, named CryoNet (https://cryonet.ai). Three subunits (PscU, PscV, and PscW) were newly identified based on the precise match of the specific amino acid side chains with the EM density (Supplementary Fig. 6). An unidentified loop spanning the transmembrane and periplasmic region, was assigned to ALA (abbreviated as UPP). In addition, two different *c*-type cytochromes, PscX (Cabther_A2183, residues 16–160 out of 189) and PscY (Cabther_A2184, residues 15–49 and 58–218 out of 221), could also be distinguished from thecryo-EM maps.

CryoNet cannot identify ligands, and the ligands from the green sulfur bacterial reaction center (GsbRC, PDB ID: 6M32) were initially docked into the 2.22 Å resolution map of CabRC_S_ using UCSF Chimera/X^49,50^. The side chains of residues and the ligands were manually deleted or added based on the cryo-EM map density using Coot^51^.

To build the CabRC_L_ model, the CabRC_S_ model was first docked into the 2.61 Å resolution EM map using UCSF Chimera. Compared to CabRC_S_, CabRC_L_ had two additional subunits, PscB (Cabther_A2187) and PscZ (Cabther_A1475). The models of PscB from the GsbRC (PDB ID: 6M32) and PscZ predicted by AlphaFold2^52^ were manually docked into the 2.61 Å resolution cryo-EM map. Model modifications were performed as described in the previous section.

All the models were finally refined with “phenix.real_space_refine” in PHENIX^53^. The refined models were corrected again in Coot until there were no more improvements in MolProbity score and geometry parameters. The final statistics for data processing and structure refinement are summarized in Supplementary Table 1.

### Reporting summary

Further information on research design is available in the Nature Portfolio Reporting Summary linked to this article.

## Supplementary information

Supplementary information

Reporting Summary

## Data Availability

The data that support this study are available from the corresponding authors upon request. Atomic coordinates have been deposited in the Protein Data Bank (PDB) under accession numbers of 7VZG and 7VZR . Cryo-EM maps have been deposited in the Electron Microscopy Data Bank (EMDB) under accession codes of EMD-32228 and EMD-32229. The source data underlying Supplementary Fig. 1a, b is provided as a Source Data file. Source data are provided with this paper.

## Source data

Source Data
