# Supplementary material for: Structure of the Acidobacteria homodimeric reaction center bound with cytochrome c
Source: Nat Commun. 2022 Dec 14;13:7745. doi: 10.1038/s41467-022-35460-6 (PMC9751088; doi:10.1038/s41467-022-35460-6)
Supplement: Supplementary file 1 — Supplementary information [file 41467_2022_35460_MOESM1_ESM.pdf]

Supplementary Information

Structure of the Acidobacteria homodimeric reaction center bound with cytochrome *c*

Shishang Dong[1], Guoqiang Huang[2], Changhui Wang[1], Jiajia Wang[1], Sen-Fang Sui[2,3*], Xiaochun Qin[1*]

[1]School of Biological Science and Technology, University of Jinan, Jinan, China.

[2]State Key Laboratory of Membrane Biology, Beijing Advanced Innovation Center for Structural Biology, School of Life Sciences, Tsinghua University, Beijing, China.

[3]Department of Biology, Southern University of Science and Technology, Shenzhen, China.

These authors contributed equally: Shishang Dong, Guoqiang Huang, Changhui Wang

*Correspondence and requests for materials should be addressed to S.-F.S. (email: suisf@mail.tsinghua.edu.cn) or to X.Q. (email: bio_qinxc@ujn.edu.cn)

Supplementary Fig. 1: Purification and characterization of the CabRC complex.

Supplementary Fig. 2: Cryo-EM data and map quality of the $CabRC_S$ complex.

Supplementary Fig. 3: Cryo-EM data processing and map quality of the $CabRC_L$ complex.

Supplementary Fig. 4: Representative cryo-EM maps of the newly identified subunits.

Supplementary Fig. 5: Comparison of the CabRC core with other representative type I and type II RC cores.

Supplementary Fig. 6: Comparison of the distribution of low-molecular-weight subunits in homodimeric type I RCs and PSII viewed from the cytoplasmic side.

Supplementary Fig. 7: The interactions between PscB and PscZ.

Supplementary Fig. 8: Representative cryo-EM maps of the key cofactors.

Supplementary Fig. 9: Comparison of the distributions of (B)Chls, carotenoids and lipids in the CabRC, GsbRC, HbRC, PSI and PSII viewed along the membrane normal from the cytoplasmic side.

Supplementary Table 1: Cryo-EM data collection, refinement and validation statistics.

Supplementary Table 2: Sequences of all subunits in $CabRC_L$.

Supplementary Table 3: Hydrogen bonds between PscX and PscY and between the PscX–PscY subcomplex and the CabRC core in the membrane-extrinsic region.

Supplementary Table 4: Hydrogen bonds and salt bridges between PscBs or PsaC and their RC cores among different type I RCs.

The uncropped gel photo of the panel b in Supplementary Fig. 1.

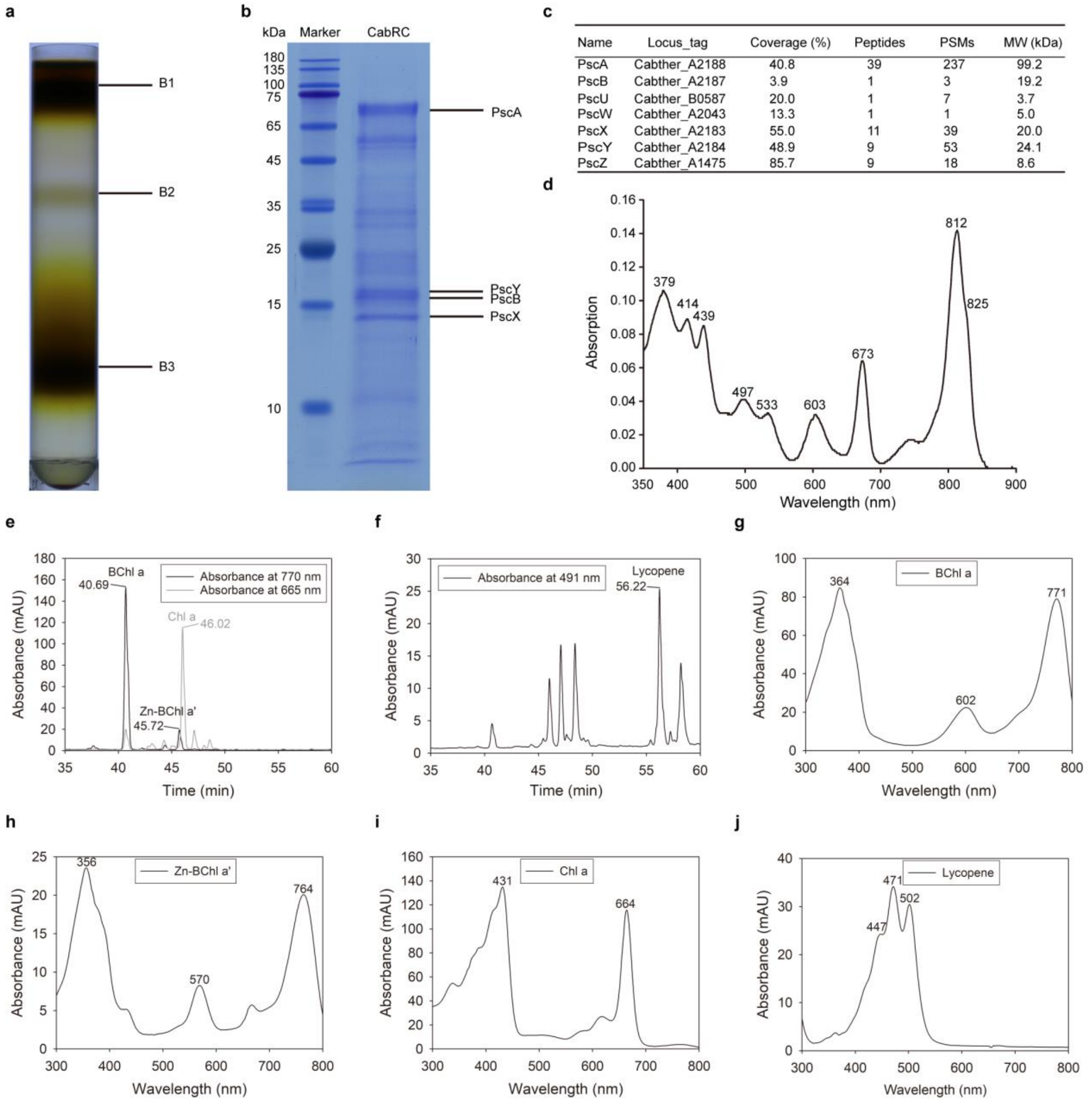

Name	Locus_tag	Coverage (%)	Peptides	PSMs	MW (kDa)
PscA	Cabther_A2188	40.8	39	237	99.2
PscB	Cabther_A2187	3.9	1	3	19.2
PscU	Cabther_B0587	20.0	1	7	3.7
PscW	Cabther_A2043	13.3	1	1	5.0
PscX	Cabther_A2183	55.0	11	39	20.0
PscY	Cabther_A2184	48.9	9	53	24.1
PscZ	Cabther_A1475	85.7	9	18	8.6

Supplementary Fig. 1: Purification and characterization of the CabRC complex. **a**, Sucrose density gradient (0.3–0.9 M) used to prepare a crude CabRC fraction. Band B2 mainly corresponds to crude CabRC samples. **b**, SDS–PAGE analysis of the B2 band purified using a Superose 6 Increase 10/300 GL column (GE). **c**, Mass spectrometric analysis of the purified CabRC using a Superose 6 Increase 10/300 GL column. **d**, Absorption spectrum of the purified CabRC complex at room temperature. **e**–**j**, HPLC analysis of pigment content in CabRC samples. HPLC chromatogram of CabRC samples showing elution peaks of BChl *a* and Zn-BChl *a*′ (detected at 770 nm) and Chl *a* (detected at 665 nm) (**e**). HPLC chromatogram of CabRC samples showing an elution peak of lycopene (detected at 491 nm) (**f**). The pigment peaks were identified based on the characteristic absorption spectrum and elute time of each fraction. **g**–**j**, Absorption spectra of BChl *a*, Zn-BChl *a*′, Chl *a* and lycopene in the HPLC eluate shown in (**g**), (**h**), (**i**) and (**j**), respectively. Collection of the data shown in this figure was repeated more than three times and always yielded the same results. Source data are provided as a Source Data file.

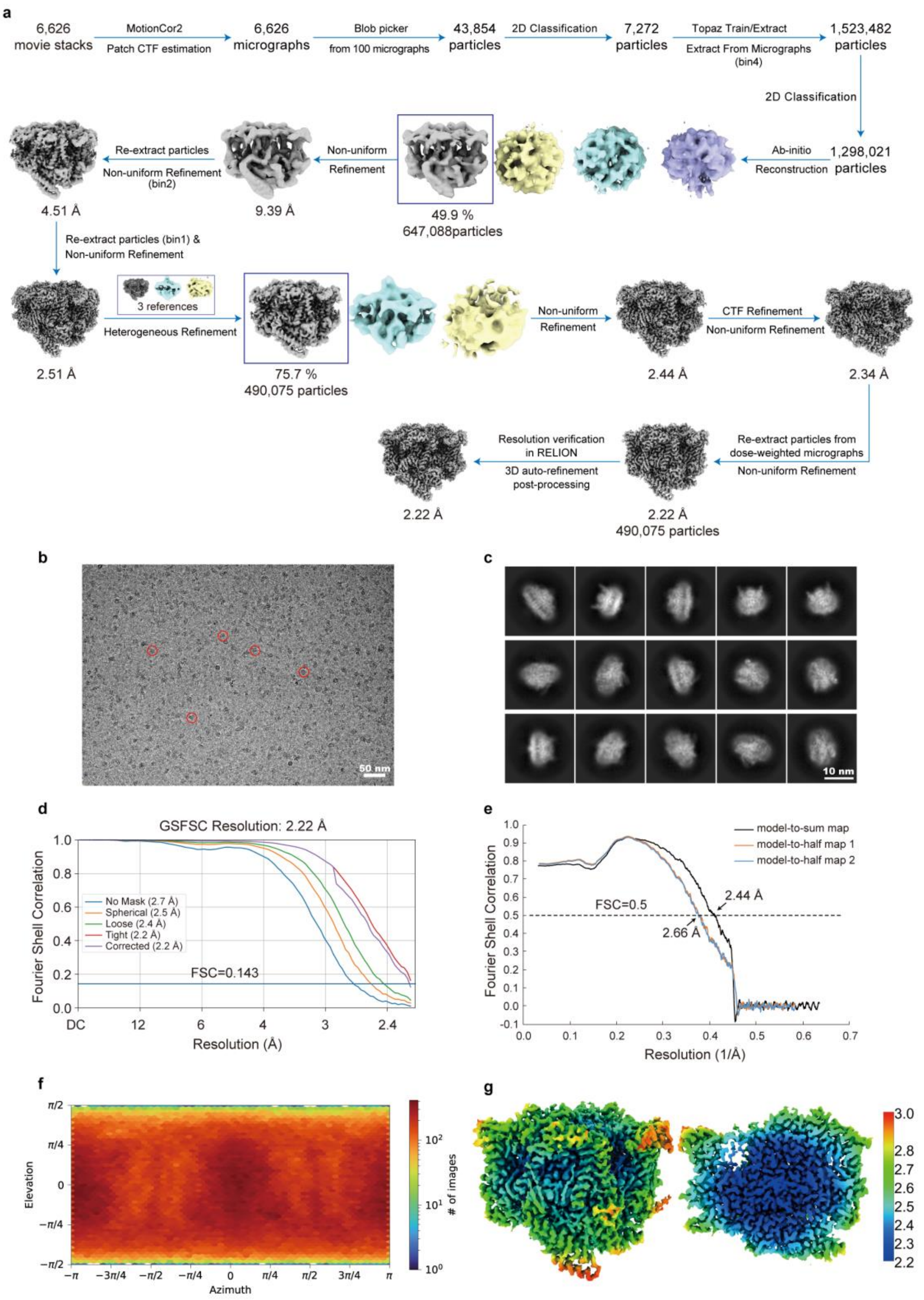

Supplementary Fig. 2: Cryo-EM data and map quality of the CabRC$_S$ complex. a, Flowchart of the cryo-EM data processing for the CabRC$_S$ complex. **b**, A representative cryo-EM micrograph of the CabRC$_S$ complex with typical particles marked by red circles. The image represents reproducible results in >90% of the collected micrographs. **c**, Representative 2D-averaged classes of the CabRC$_S$ complex. **d**, Gold-standard Fourier shell correlation (FSC = 0.143) curves of the CabRC$_S$ complex. The unmasked, spherical, loose, tight and corrected curves are shown in different colors. **e**, Model versus map FSC curves for the CabRC$_S$ complex. FSC curves of the final refined model of the CabRC$_S$ complex versus the sum map (black).

Half map 1 (orange) and half map 2 (blue) of the two independent half maps were calculated by PHENIX. The similarity between the orange and blue curves indicates that the refinement of the atomic coordinates did not suffer from over-fitting. **f**, Angular distribution of the particles used for final reconstruction of the $CabRC_S$ complex. **g**, Local resolution distribution of the $CabRC_S$ complex calculated with RELION3.1, the cryo-EM map was sharpened and shown at a contour level of 0.48.

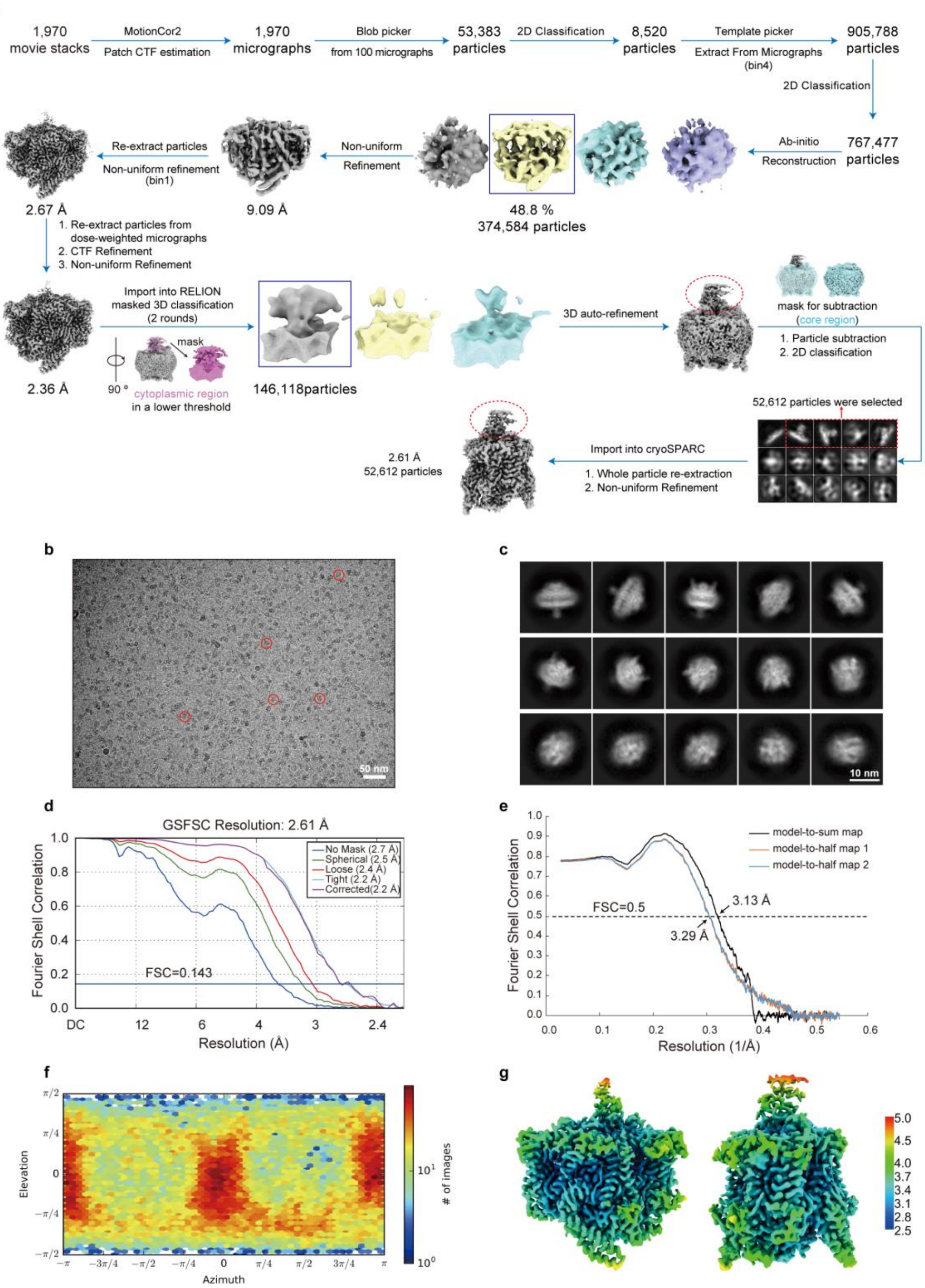

Supplementary Fig. 3: Cryo-EM data processing and map quality of the CabRC$_L$ complex. **a**, Flowchart of the cryo-EM data processing for the CabRC$_L$ complex. **b**, A representative cryo-EM micrograph of the CabRC$_L$ complex with typical particles marked by red circles. The image represents reproducible results in >90% of the collected micrographs. **c**, Representative 2D-averaged classes of the CabRC$_L$ complex. **d**, Gold-standard Fourier shell correlation (FSC=0.143) curves of the CabRC$_L$ complex. The unmasked, spherical, loose, tight and corrected curves are shown with different colors. **e**, Model versus map FSC curves for the CabRC$_L$ complex. FSC curves of the final refined model for the CabRC$_L$ complex versus the

sum map (black). Half map 1 (orange) and half map 2 (blue) of the two independent half maps were calculated by PHENIX. The similarity between the orange and blue curves indicates that the refinement of the atomic coordinates did not suffer from over-fitting. **f**, Angular distribution of the particles used for final reconstruction of the CabRC_{L} complex. **g**, Local resolution maps for the CabRC_{L} complex calculated with RELION3.0, the cryo-EM map was sharpened and shown at a contour level of 0.35.

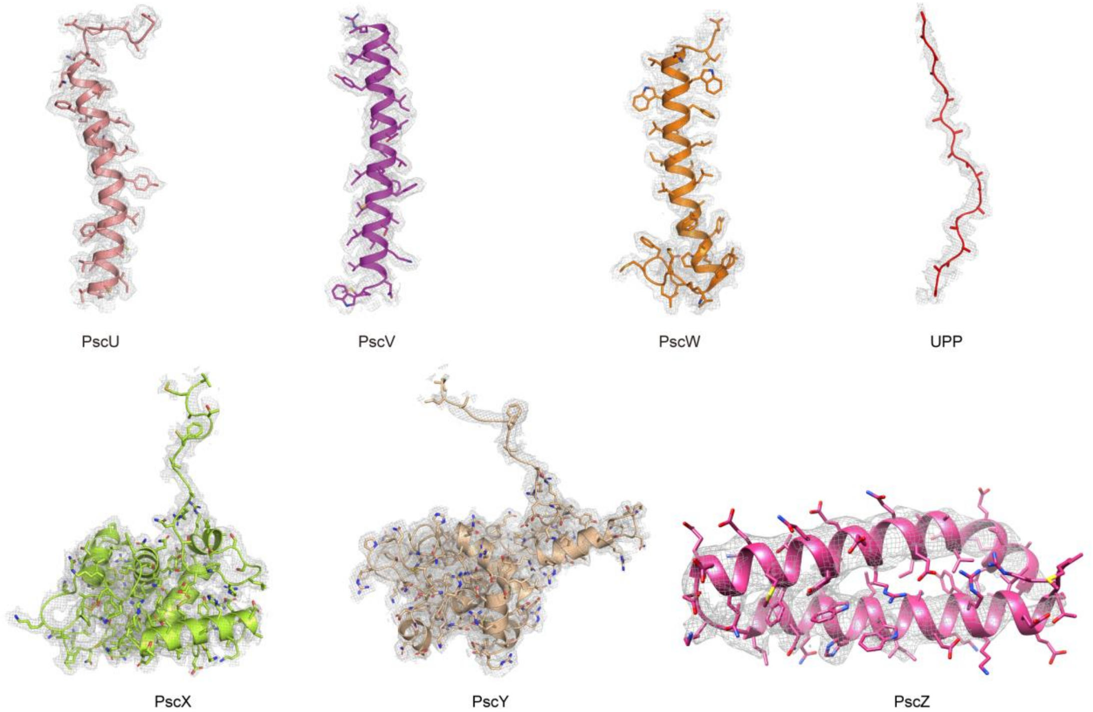

Supplementary Fig. 4: Representative cryo-EM maps of the newly identified subunits. PscU: salmon red; PscV: magenta; PscW: brown; PscX: lemon; PscY: wheat; PscZ: hot pink; UPP: red; all subunits are shown as cartons, and all densities are shown as grey meshes. All the cryo-EM maps were sharpened and shown at a contour level of 0.48, except for PscZ, which was shown at a contour level of 0.25.

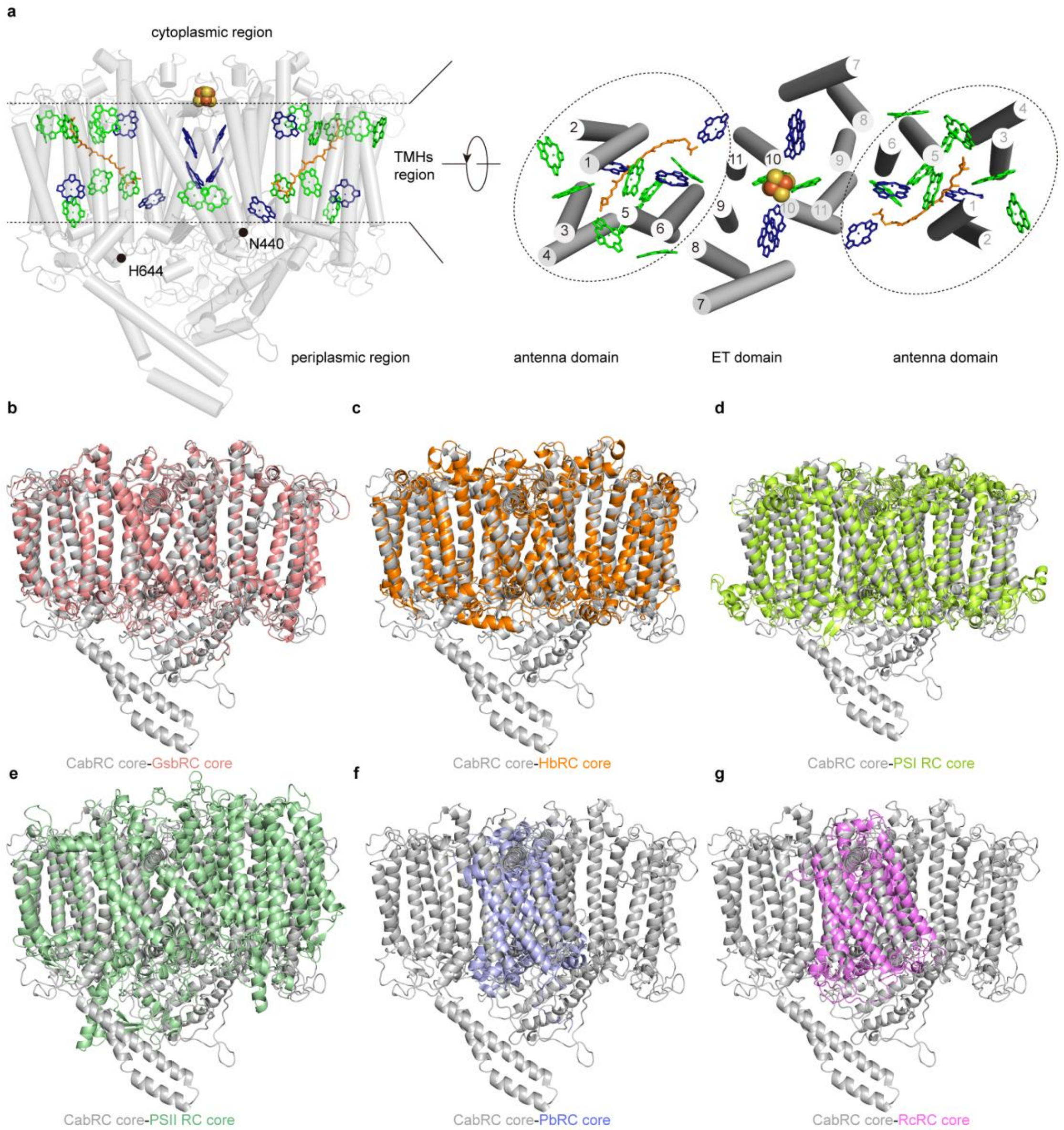

Supplementary Fig. 5: Comparison of the CabRC core with other representative type I and type II RC cores. **a**, Structure of the CabRC core (PscA-1–PscA-2 homodimer). The cytoplasmic region, periplasmic region and TMH region are indicated, and the TMH region is divided into two antenna domains and one ET domain. TMHs (1–11) are numbered from the N to the C terminals. Chls *a* are colored dark blue, Zn-BChls *a*′ and BChls *a* are colored green, and lycopenes are colored brown. **b** to **d**, Superposition of the CabRC core with other representative type I RC cores, i.e., the GsbRC core (PscA homodimer, PDB code: 6M32) (**b**), the HbRC core (PshA homodimer, PDB code: 5V8K) (**c**) and a PSI RC core (PsaA and PsaB, PDB code: 1JB0) (**d**). **e** to **g**, Superposition of the CabRC with representative type II RC cores, i.e., a PSII RC core (D1, D2, CP43 and CP47, PDB code: 3WU2) (**e**), the PbRC core (L subunit and M subunit, PDB code: 5Y5S) (**f**) and the RC core from *Roseiflexus castenholzii* (L subunit and M subunit, termed RcRC core, PDB code: 5YQ7) (**g**).

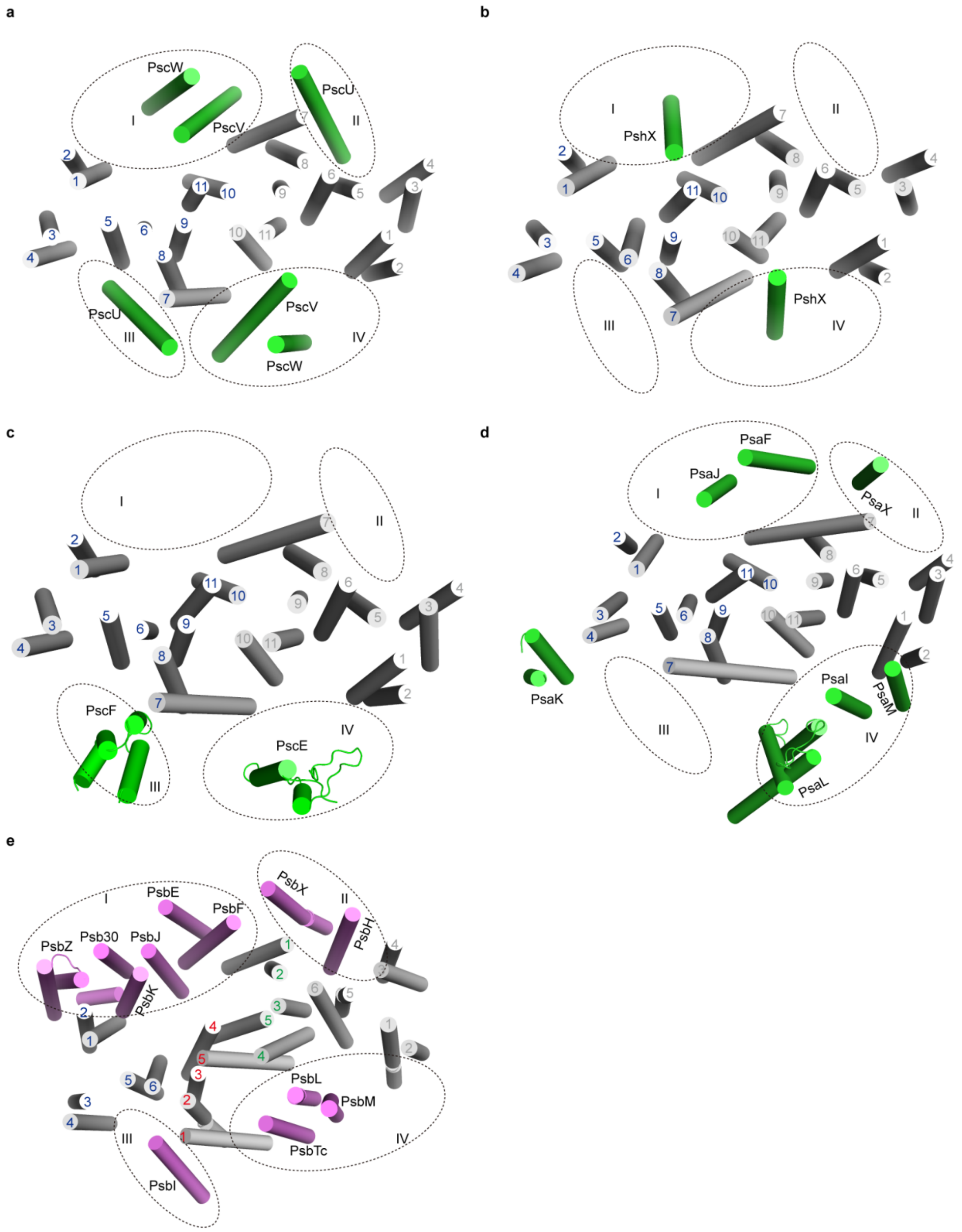

Supplementary Fig. 6: Comparison of the distribution of low-molecular-weight subunits in homodimeric type I RCs and PSII viewed from the cytoplasmic side. **a** to **d**, The distribution of low-molecular-weight subunits in the CabRC (**a**), HbRC (**b**), GsbRC (**c**) and PSI (**d**). RC cores are shown as cartoons and colored grey, and low-molecular-weight subunits are arranged in clusters I–IV. TMHs (numbers 1–11) of the largest subunits are depicted in blue and grey for each side. Low-molecular-weight subunits are colored green. **e**, The distribution of low-molecular-weight subunits in PSII. Low-molecular-weight proteins are colored magenta. TMHs of CP43 (numbers 1–6) and CP47 (numbers 1–6) are depicted in blue and grey, respectively, and TMHs of D1 (numbers 1–5) and D2 (numbers 1–5) are

depicted in red and green, respectively. The PDB codes of all models expect for GsbRC (PDB: 7UEB) used here are the same as in Supplementary Fig. 5.

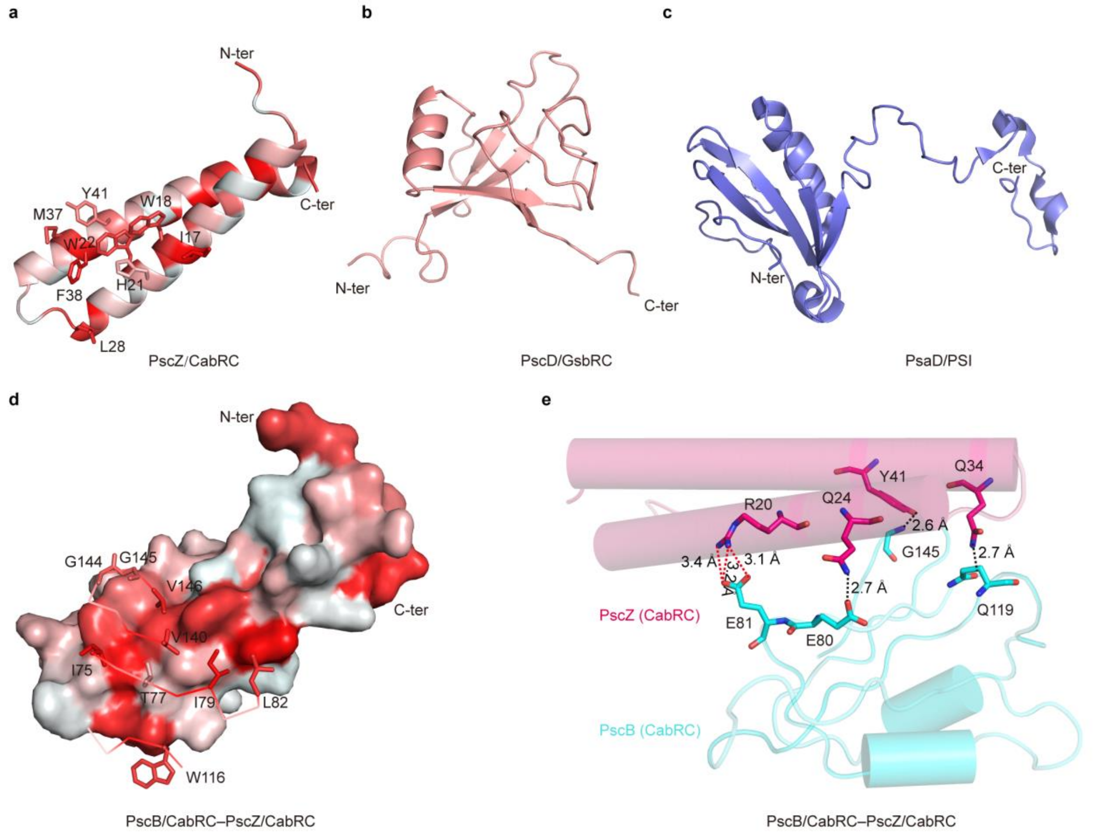

Supplementary Fig. 7: The interactions between PscB and PscZ. a, **b** and **c,** structures of PscZ in the CabRC, PscD in the GsbRC (PDB code: 7UEB) and PsaD in PSI (PDB code: 1JB0). The main chain of PscZ is shown as a cartoon and its hydrophobic residues on the interface are shown as sticks. **d**, The hydrophobic interface between PscZ and PscB. PscZ is shown as a surface, and the hydrophobic residues of PscB on the interface are shown as sticks. All residues of PscZ in (**a**) and (**d**) are shaded red according to their hydrophobicity. **e**, Hydrogen bonds (black dotted lines) and salt bridges (red dotted lines) formed at the interface between PscZ and PscB.

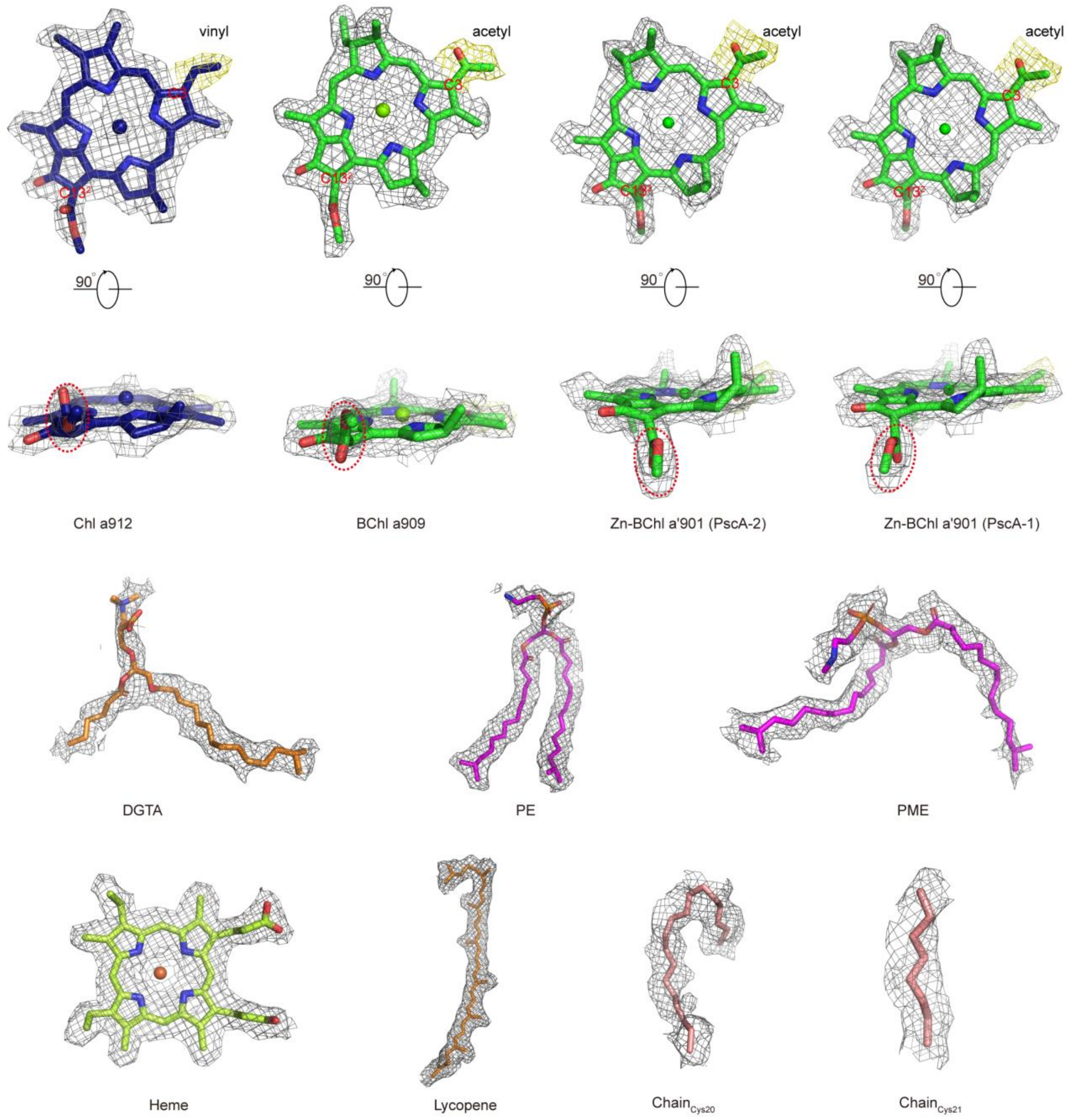

Supplementary Fig. 8: Representative cryo-EM maps of the key cofactors. Chl *a*: sticks, dark blue; BChl *a* and Zn-BChl *a′*: sticks, green; PE and PME: sticks, magenta; Heme: sticks, lemon; DGTA and Lycopene: sticks, brown; Chain$_{Cys20}$ and Chain$_{Cys21}$: sticks, salmon red; all densities are shown as grey meshes. One difference (the presence of vinyl or acetyl at the C3 of the tetrapyrrole ring) between Chl *a* and BChl *a* (or Zn-BChl *a′*) is indicated by the yellow region in the maps. One difference (an epimer exhibiting reversed stereochemistry at the 13^2 carbon of the tetrapyrrole ring) between BChl *a* and Zn-BChl *a′* is marked with a red dotted oval. All the cryo-EM maps were sharpened and shown at a contour level of 0.48.

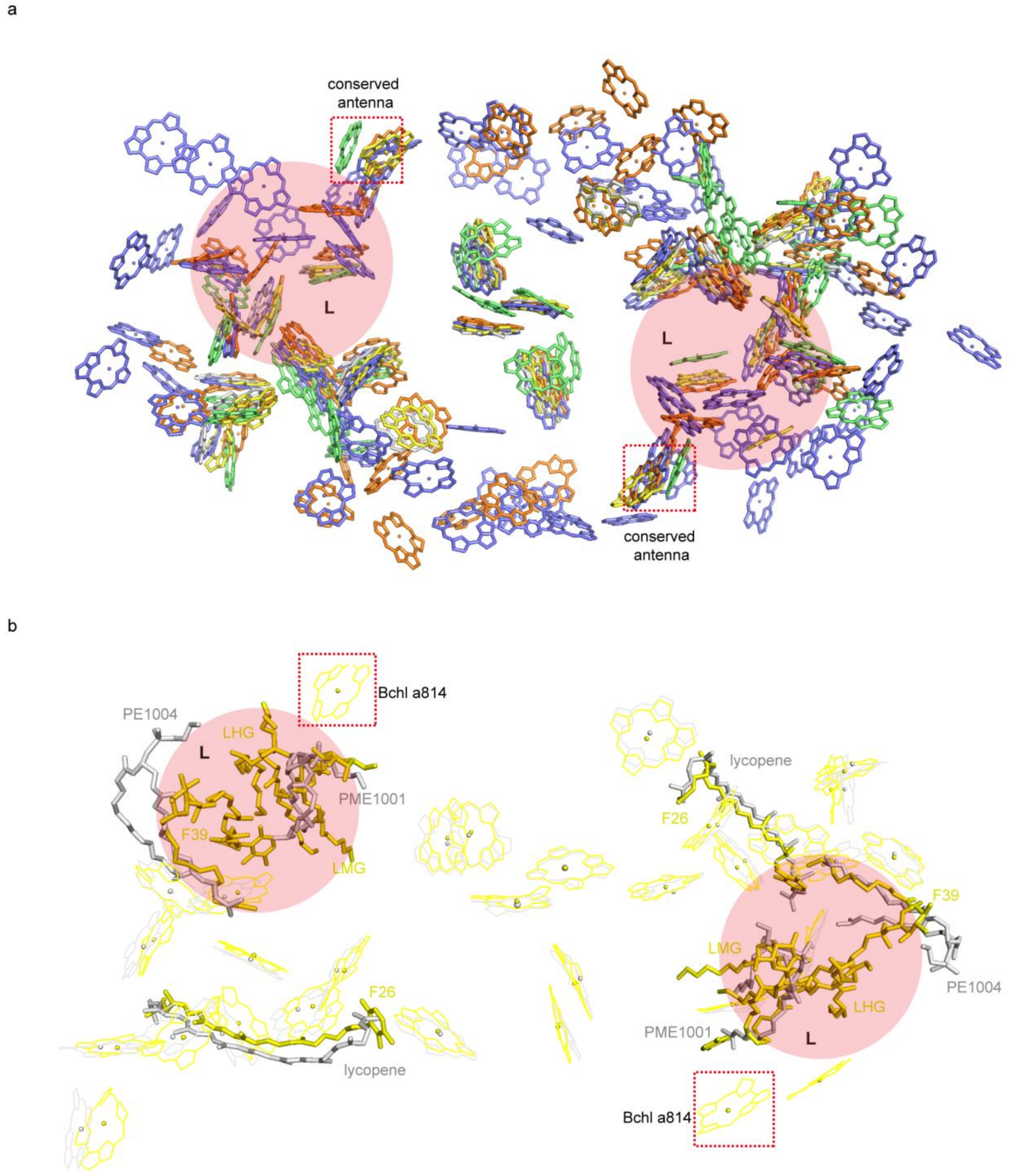

Supplementary Fig. 9: Comparison of the distributions of (B)Chls, carotenoids and lipids in the CabRC, GsbRC, HbRC, PSI and PSII viewed along the membrane normal from the cytoplasmic side. a, Comparison of the distributions of (B)Chls in the CabRC, GsbRC, HbRC, PSI and PSII. The (B)Chls in the CabRC, GsbRC, HbRC, PSI and PSII are colored gray, yellow, brown, blue and green, respectively. **b**, Comparison of the distributions of carotenoids and lipids (shown as stick models) in the "L" regions from the CabRC and GsbRC. Only the main tetrapyrrole rings are shown for (B)Chls, and they are shown as line models. The "L" regions are shaded with red circles. The conserved antennas are indicated by red dotted boxes. The PDB codes of all models used here are the same as in Supplementary Fig. 6.

Supplementary Table 1: Cryo-EM data collection, refinement and validation statistics.

	#1 $CabRC_S$ (EMDB-32229) (PDB 7VZR)	#2 $CabRC_L$ (EMDB-32228) (PDB 7VZG)
**Data collection and processing**		
Magnification	81,000 ×	81,000 ×
Voltage (kV)	300	300
Electron exposure (e^{-}/Å^2)	50	50
Defocus range (μm)	-1.0 to -1.5	-1.0 to -1.5
Pixel size (Å)	1.0825	1.0825
Symmetry imposed	C1	C1
Initial particle images (no.)	1,523,482	905,788
Final particle images (no.)	490,075	52,612
Map resolution (Å)	2.22	2.61
FSC threshold	0.143	0.143
Map resolution range (Å)	2.2 - 3.0	2.5 - 5.0
**Refinement**		
Initial model used (PDB code)	6M32	$CabRC_S$
Model resolution (Å)	2.22	2.61
FSC threshold	0.143	0.143
Model resolution range (Å)	n/a	n/a
Map sharpening *B* factor (Å^2)	-74.7	-64.7
Model composition		
Non-hydrogen atoms	21,373	22,346
Protein residues	2,307	2,427
Ligands	79	81
B factors (Å^2)		
Protein	27.70	125.92
Ligand	23.88	112.21
R.m.s. deviations		
Bond lengths (Å)	0.009	0.009
Bond angles (°)	2.275	2.184
Validation		
MolProbity score	2.35	2.66
Clashscore	6.10	7.96
Poor rotamers (%)	7.81	11.73
Ramachandran plot		
Favored (%)	95.13	93.70
Allowed (%)	4.69	6.13
Disallowed (%)	0.18	0.17

Supplementary Table 2: Sequences of all subunits in CabRC$_L$.

Subunits	The primary sequences
**PscA (Cabther_A2188)**	MASFSSYANGVKRWYQKLELPMPPERIFGAHMMLIGGLACLIGTYFFAS<br>MTMWNDGYVNLTLRPRLISLGIYDPYDTEQIQRVWLPLIGEFSTSKLPFF<br>GQYPLTMTDFRLFGWGCFHIGLGLWLVYAGAAHYYGARGGATIGEIFW<br>LLPYVPGLKGLCQIKWFTPEGPWYKVGLPWGSFANTPWPILRRTYADAL<br>SPHTIYIGLLFFIWGFVLWFVLDKPPVPLQPAQVMTPNGLMPLEQAPFPY<br>GWFDPYLNQVMHPMNTINGETTMCFVWGVLFVALGAYWWYRPPRSIN<br>ITHLEDTKAVFHVHLTAIGYVSFALAIVGFLALRNHPSYLMLNDMNVIIY<br>GKKIVNPGRMIHNMITFNHVQVGLLYVAAGVFHGGQYLHGLNISGAYK<br>QARSKFITWFQNPDLQTKIVGTTMFVSFVTVVFGYGMICWNTGAELDLN<br>FGIYQFRSFRAIQMDGEAGNIGYRVFRPKNPWDPTAGGDWVKNPDGTA<br>KLVKARNLQVGDRILNEELGIGSSPTYSFTTIEEINYKPEWGQPKLYAVQ<br>WGSWTHFLRKVNPLFWVDKGIWYLQNQKTFEATRKADEAYLAAHLKA<br>VSLLNQIDDAQTEEAKQKAQAELDKFRPELEKAHANMLEWNERLASTP<br>AVLYSNLRDQHRDGEINDAIFFWLMIGGWLFGFIPLLRIAFHNYQSPWY<br>RDFEWRKQSPDFPCIGPVKGGTCGVSIQDQLWFCILFSIKPLSAIAWYLD<br>GGWIATMMARGNEAYYLTHNISHTGGVFLYMWNETTWIWTDNHLTA<br>MLLLGHLIWFVSFALWFKDRGSRAEGGDIQSRWVRLMGKRLGIKTLQE<br>VRFPVSNLATAKLWGTVFFYTGTFVLVFLYFADGFFQNR
**PscB (Cabther_A2187)**	MKGKVTMAAEEKAETTNGAPAETPALAPAKAAAKAAAKAPAKAAAK<br>AAPPPIPGPPSRLSRPKTAAPKKKRERQIYTIIEELCIGCGFCTDECPPKVN<br>AILPRDVEAVLDGGETYWIDQTRCISCSLCFVAGTCPTDAVVFTEGGVSR<br>TQYMEDYLHIEMVDEPYWRQRNELSRIGSIL
**PscU (Cabther_B0587)**	MTAILLACLFVLGGYAALWGIIKFVVANTKDIAAN
**PscV (Cabther_A1265)**	MWNVVGQIISVLCFFILTVGTLFGIVYVSHLLSRGQ
**PscW (Cabther_A2043)**	MEGVAMEDISKVAWAWFGVLLAICLIGAFGNYVPKLFVKMLMFLN
**PscX (Cabther_A2183)**	MTDKVKTVALLGLTAVMATGCFVGARNASEPRLGSSSIAASRTAPAYL<br>REAQVLYEGSTDGLPKDTPADEIAHYKAMLAELQTRNYAACAGCHQV<br>NGGGNKAINATNFQDAGWQANNSSPGMVTSIVNGKGKVMPAYKDKLT<br>LQQINYLVEYIRRFEKKRTDAAPITAGIPSGTTTPAELPVAEATAQR
**PscY (Cabther_A2184)**	MKSIKIIVLGSALALLVGGCFVGSRDPNETRYPKAPMPLQNQTSTLKTAE<br>EIRRESVAQNTPGAREAAALRDRVTPLNLQQVNEQDVAGNDPLGSPAR<br>VVLDEGEMYRDPVEIYREGRALFQNNCVGCHGHNGCGNVPRSTNFTDP<br>GWQENNSDGGIYSSIYNGKGIGNGGGAMPAYYNQLSPQQIRYLVAYLR<br>AFKGRQCNGLPTLSDVERMVAERQNKP
**PscZ (Cabther_A1475)**	MARTPEEIVKRYKEANIWLRHWKQQIGLAKDEEQREMFTQYYEERVQE<br>IAALEEPYRAALKILNQQESQR
**UPP**	AAAAAAAAAAAAAAAAAAA

Supplementary Table 3: Hydrogen bonds between PscX and PscY and between the PscX–PscY subcomplex and the CabRC core in the membrane-extrinsic region.

NO.	Subnit 1	Subunit 2	Dis. (Å)	NO.	Subunit 1	Subunit 2	Dis. (Å)
	**PscX**	**PscY**			**PscA-2**	**PscX**	
1	ASN 117[OD1]	LYS 165[NZ]	3.35	1	GLU 505[OE1]	LEU 143[N]	3.26
2	LYS 132[NZ]	PRO 96[O]	2.65		**PscA-2**	**PscY**	
3	LYS 132[NZ]	VAL 99[O]	2.88	1	ASN 54[N]	GLY 23[O]	2.76
4	GLY 131[N]	SER 159[OG]	2.83	2	ASN 54[ND2]	SER 24[O]	3.36
5	THR 124[OG]	LYS 165[O]	2.39	3	LYS 346[NZ]	GLU 29[OE1]	3.78
6	LYS 130[NZ]	ASN 152[OD1]	2.95	4	LYS 346[NZ]	GLU 29[OE2]	3.42
7	ASP 110[OD1]	ASN 151[ND2]	3.25	5	ARG 865[NH2]	MET 37[O]	3.74
8	ASN 117[OD1]	ARG 139[NH1]	2.58	6	ARG 865[NH2]	PRO 38[O]	3.17
9	GLN 114[O]	ARG 139[NH1]	3.86	7	ARG 641[NH2]	LEU 39[O]	3.89
10	ASN 116[O]	THR 141[OG1]	3.20	8	ARG 641[NE]	LEU 39[O]	3.83
11	ALA 115[O]	ARG 139[NH2]	2.98	9	ARG 641[NH2]	ASN 41[OD1]	2.87
	**PscA-1**	**PscX**		10	THR 77[OG1]	THR 61[OG1]	3.90
1	ASN 54[ND2]	VAL 23[O]	3.00	11	ARG 865[N]	VAL 82[O]	2.98
2	ASN 54[N]	GLY 24[O]	2.86	12	ILE 753[N]	GLU 84[OE1]	3.58
3	GLN 79[N]	GLY 4[O]	3.43	13	ASN 864[ND2]	GLU 84[OE2]	3.20
4	LYS 346[NZ]	ASN 147[O]	3.72	14	TRP 769[NE1]	ASN 124[OD1]	3.42
5	LYS 346[NZ]	GLU 151[OE1]	3.21	15	ARG 742[NE]	ASN 169[O]	3.08
6	THR 44[OG1]	GLY 20[N]	3.30	16	ASN 744[ND2]	ALA 176[O]	3.15
7	THR 51[O]	ARG 42[NH1]	3.06	17	ALA 746[N]	TYR 178[OH]	3.75
8	MET 52[O]	ARG 26[N]	2.95	18	MET 52[O]	ARG 25[N]	2.84
9	TRP 53[O]	ARG 42[NH1]	3.24	19	ASN 339[O]	ARG 25[NH1]	3.03
10	TRP 53[O]	ARG 42[NH2]	3.18	20	ILE 341[O]	ARG 25[NH2]	3.87
11	ASP 55[OD1]	ARG 42[NH2]	2.23	21	ASN 339[O]	ARG 25[NH2]	2.94
12	ASP 55[OD2]	ARG 26[NH2]	3.34	22	ASP 55[OD2]	ARG 25[NH2]	3.00
13	THR 77[O]	SER 36[N]	3.10	23	ASP 55[OD1]	ARG 31[NE]	3.21
14	ASP 337[O]	TYR 47[OH]	2.96	24	THR 51[O]	ARG 31[NH1]	3.02
15	MET 338[O]	TYR 47[OH]	3.41	25	TRP 53[O]	ARG 31[NH2]	2.77
16	ASN 339[O]	ARG 26[NH2]	2.95	26	ASP 55[OD1]	ARG 31[NH2]	3.10
17	ASN 339[O]	ARG 26[NH1]	3.33	27	ASP 337[O]	LYS 34[N]	3.27
18	ASN 339[OD1]	ARG 26[NH1]	3.44	28	ASP 337[OD1]	LYS 34[NZ]	3.68
19	ASN 339[OD1]	GLN 144[NE2]	3.49	29	GLU 627[OE1]	SER 44[OG]	2.71
20	GLU 627[OE2]	LYS 159[NZ]	3.38	30	GLU 627[OE2]	THR 45[N]	3.30
	**PscA-1**	**PscY**		31	GLU 627[OE2]	LEU 46[N]	3.69
1	ASN 744[N]	GLY 135[O]	3.11	32	LEU 749[O]	ASN 90[ND2]	3.63
2	ASN 765[ND2]	ASN 136[OD1]	3.15	33	ASN 752[OD1]	ASN 90[ND2]	3.28
3	TRP 74[NE1]	PRO 138[O]	2.94	34	LYS 346[O]	ASN 123[ND2]	3.62
4	THR 768[OG1]	ALA 173[O]	3.70	35	TRP 771[O]	HIS 131[NE2]	3.89
5	GLN 453[N]	GLN 200[OE1]	3.28	36	SER 511[OG]	ASN 169[ND2]	3.39

6	ALA 451[N]	ASN 202[OD1]	3.58	37	GLU 45[OE2]	GLY 170[N]	3.51
7	GLY 508[O]	ARG 118[NH1]	3.21	38	ASN 744[OD1]	TYR 178[N]	3.58
8	GLY 508[O]	ARG 118[NH2]	3.22	39	ASN 744[OD1]	ASN 179[N]	2.84
9	ASN 744[O]	ASN 132[ND2]	2.89	40	GLY 743[O]	ASN 179[ND2]	3.08
10	ASN 765[OD1]	ASN 136[ND2]	3.33	41	ILE 753[O]	ASN 179[ND2]	3.13
				42	ASN 744[OD1]	GLN 180[N]	3.42

Supplementary Table 4: Hydrogen bonds and salt bridges between PscBs or PsaC and their RC cores among different type I RCs.

Bonding	NO.	Subnit 1	Subunit 2	Dis. (Å)
		**PscB/CabRC**	**PscA-2/CabRC**	
Hydrogen bonds	1	ASP 91[OD1]	LYS 701 [N]	3.73
		**PscB/GsbRC**	**PscA-1/GsbRC**	
Hydrogen bonds	1	ARG 215[NH1]	ASN 405[O]	3.64
	2	PRO 192[O]	LYS 519[N]	3.08
	3	CYS 191[O]	ASN 521[ND2]	3.60
	4	ASP 189[O]	ASN 521[ND2]	3.88
	5	GLU 190[O]	ASN 521[ND2]	3.70
	6	GLN 166[O]	TYR 532[N]	2.97
	7	GLN 166[O]	GLY 533[N]	3.35
		**PscB/GsbRC**	**PscA-2/GsbRC**	
Hydrogen bonds	1	CYS 143[O]	VAL 531[N]	3.79
	2	ASP 148[OD1]	TYR 532[N]	3.49
		**PsaC/PSI**	**PsaA/PSI**	
Hydrogen bonds	1	ARG 52[NH2]	ASP 568[OD1]	2.84
	2	ARG 52[NH1]	ASP 568[OD2]	3.22
	3	ARG 52[NH2]	ASP 579[OD1]	2.77
	4	ARG 52[NE]	ASP 579[OD2]	3.07
	5	VAL 48[O]	ARG 583[N]	2.84
Salt bridges	1	ARG 52[NH1]	ASP 568[OD1]	3.67
	2	ARG 52[NH2]	ASP 568[OD1]	2.84
	3	ARG 52[NH1]	ASP 568[OD2]	3.22
	4	ARG 52[NH2]	ASP 568[OD2]	3.47
	5	ARG 52[NE]	ASP 579[OD1]	3.04
	6	ARG 52[NH2]	ASP 579[OD1]	2.77
	7	ARG 52[NE]	ASP 579[OD2]	3.07

	8	ARG 52[NH2]	ASP 579[OD2]	3.93
		**PsaC/PSI**	**PsaB/PSI**	
Hydrogen bonds	1	LYS 51[NZ]	ASP 566[OD1]	2.60
	2	ARG 65[NH1]	ASP 555[OD2]	2.98
	3	ARG 65[NH1]	ASP 555[OD1]	3.11
	4	ARG 65[NH2]	ASP 566[OD2]	2.63
	5	TYR 80[OH]	PRO 703[O]	2.67
	6	TYR 80[OH]	GLN 678[OE1]	3.18
	7	THR 73[OG1]	LYS 702[NZ]	2.96
	8	TYR 80[O]	LYS 702[NZ]	2.52
Salt bridges	1	LYS 51[NZ]	ASP 566[OD1]	2.60
	2	ARG 65[NE]	ASP 555[OD2]	3.87
	3	ARG 65[NH1]	ASP 555[OD2]	2.98
	4	ARG 65[NH1]	ASP 555[OD1]	3.11
	5	ARG 65[NH1]	ASP 566[OD2]	3.67
	6	ARG 65[NH2]	ASP 566[OD1]	3.06
	7	ARG 65[NH2]	ASP 566[OD2]	2.63

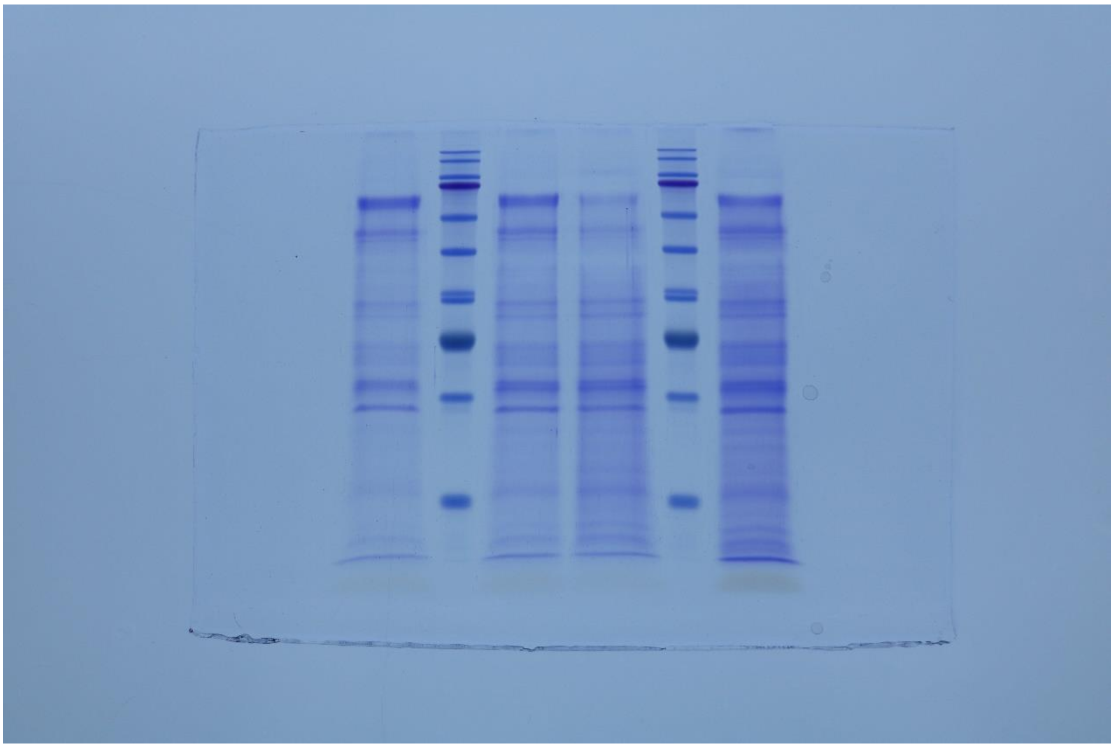

The uncropped gel photo of the panel b in Supplementary Fig. 1.
