# Supplementary figures and images for: Structure of the Acidobacteria homodimeric reaction center bound with cytochrome c
Source: Nat Commun. 2022 Dec 14;13:7745. doi: 10.1038/s41467-022-35460-6 (PMC9751088; doi:10.1038/s41467-022-35460-6)

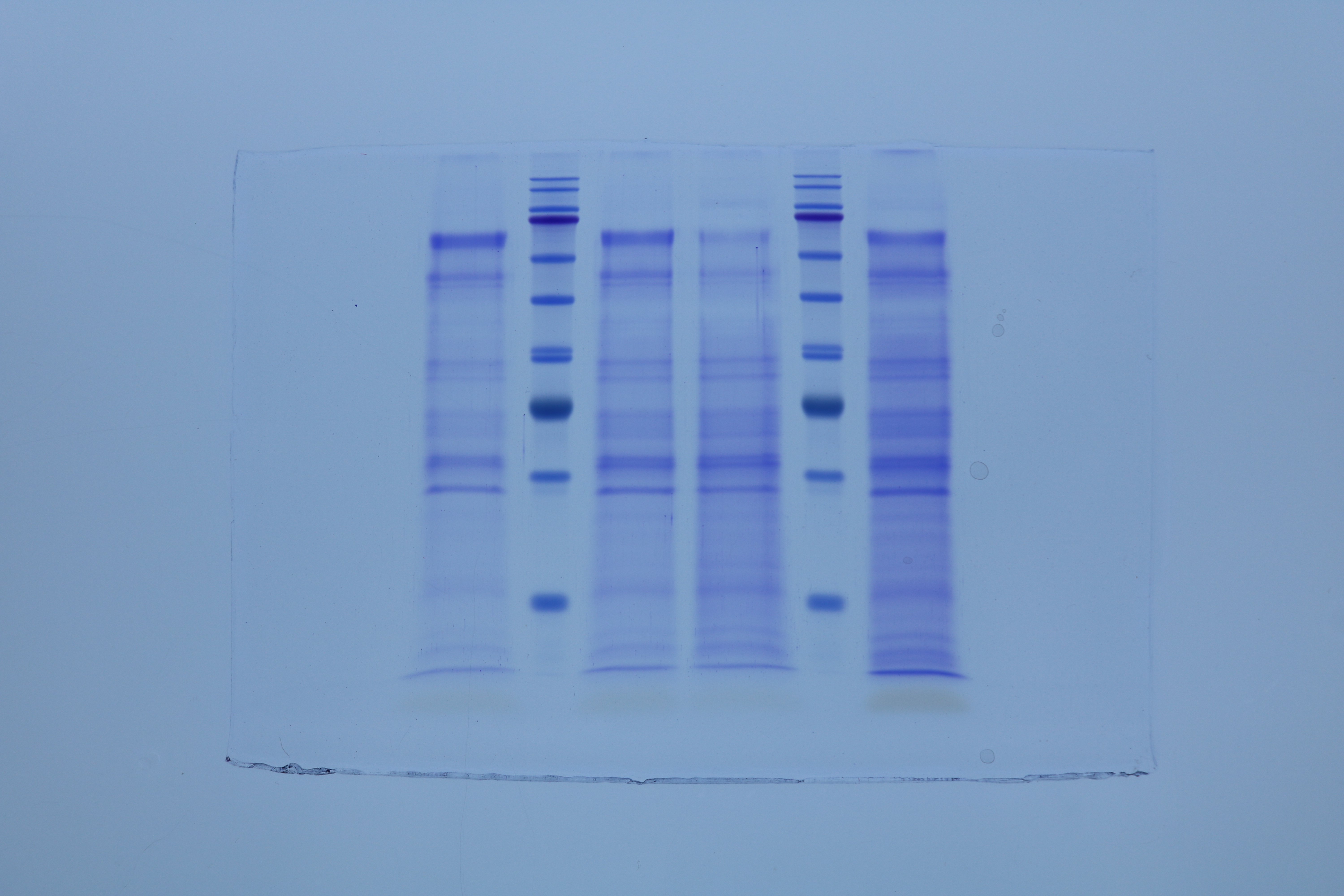

Supplement: Supplementary file 3 — Source Data [file 41467_2022_35460_MOESM3_ESM.zip › Source Data/SDS‒PAGE analysis.JPG]

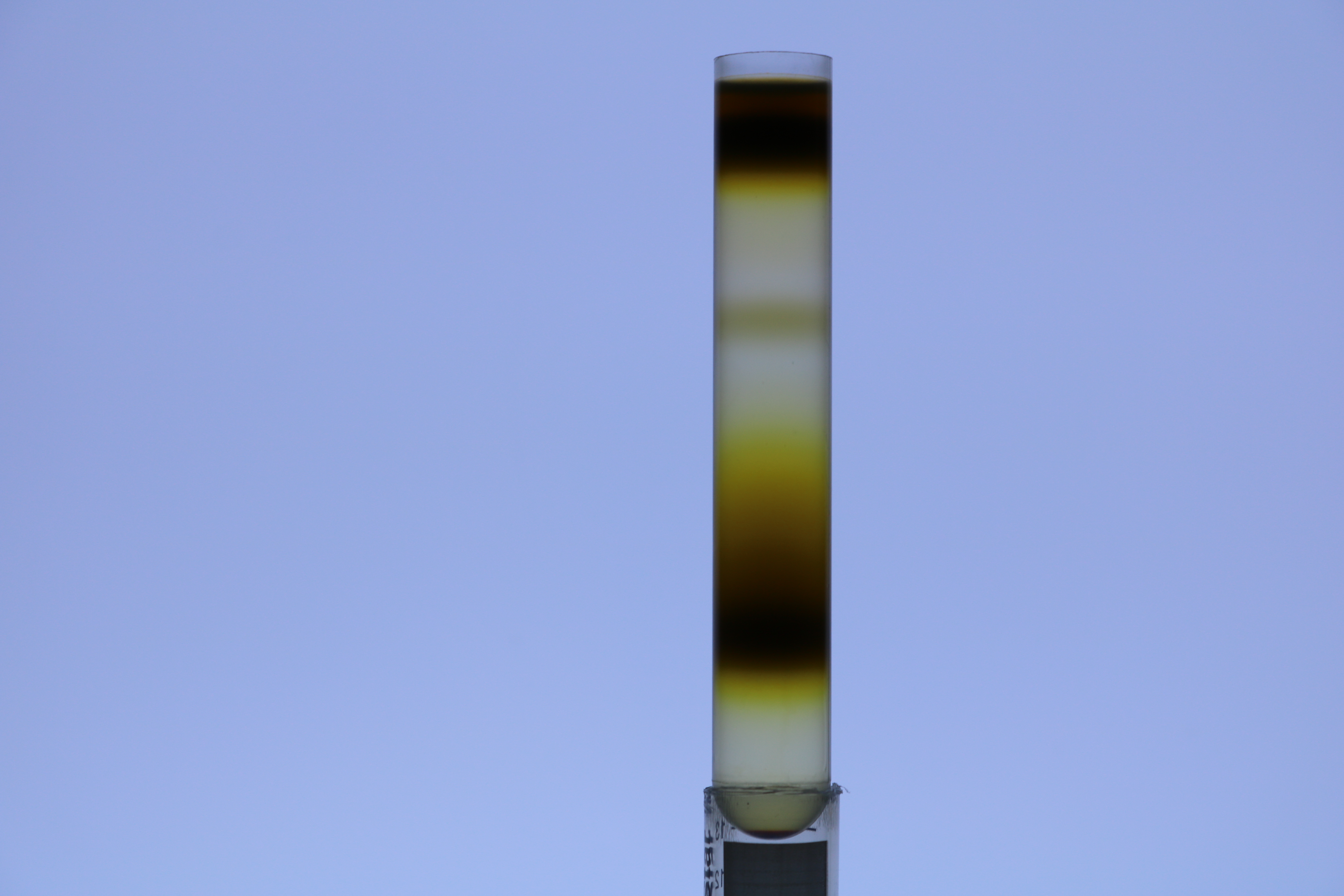

Supplement: Supplementary file 3 — Source Data [file 41467_2022_35460_MOESM3_ESM.zip › Source Data/Sucrose density gradient.jpg]
